# Blood Component Therapy and Coagulopathy in Trauma: A Systematic Review of the Literature from the Trauma Update Group

**DOI:** 10.1371/journal.pone.0164090

**Published:** 2016-10-03

**Authors:** Daniele Poole, Andrea Cortegiani, Arturo Chieregato, Emanuele Russo, Concetta Pellegrini, Elvio De Blasio, Francesca Mengoli, Annalisa Volpi, Silvia Grossi, Lara Gianesello, Vanni Orzalesi, Francesca Fossi, Osvaldo Chiara, Carlo Coniglio, Giovanni Gordini, Trauma Update Working Group

**Affiliations:** 1 Anesthesia and Intensive Care Operative Unit, “S. Martino” Hospital, Belluno, Italy; 2 Department of Biopathology and Medical Biotechnologies (DIBIMED), Section of Anesthesia, Analgesia, Intensive Care, and Emergency, Policlinico “P. Giaccone”, University of Palermo, Palermo, Italy; 3 Neurointensive Care Unit ASST Great Metropolitan “Niguarda” Hospital, Milan, Italy; 4 Anaesthesia and Intensive Care Unit, Surgical and Severe Trauma Department, “Bufalini” Hospital, Cesena, Italy; 5 Anesthesia and Intensive Care, AO “Rummo”, Benevento, Italy; 6 UOC Intensive Care and Territorial Emergency Department, “Maggiore” Hospital, Bologna, Italy; 7 Anesthesia and Intensive Care, AOU of Parma, Parma, Italy; 8 Departmental Structure of Anesthesia and Intensive Care for Orthopedic Surgery, AOU “Careggi”, Florence, Italy; 9 Neuroanesthesia and Neurointensive Care, AOU “Careggi”, CTO, Florence, Italy; 10 Trauma Center Department, ASST Great Metropolitan Niguarda Hospital, Milan, Italy; Maastricht University Medical Center, NETHERLANDS

## Abstract

**Background:**

Traumatic coagulopathy is thought to increase mortality and its treatment to reduce preventable deaths. However, there is still uncertainty in this field, and available literature results may have been overestimated.

**Methods:**

We searched the MEDLINE database using the PubMed platform. We formulated four queries investigating the prognostic weight of traumatic coagulopathy defined according to conventional laboratory testing, and the effectiveness in reducing mortality of three different treatments aimed at contrasting coagulopathy (high fresh frozen plasma/packed red blood cells ratios, fibrinogen, and tranexamic acid administration). Randomized controlled trials were selected along with observational studies that used a multivariable approach to adjust for confounding. Strict criteria were adopted for quality assessment based on a two-step approach. First, we rated quality of evidence according to the Grading of Recommendations Assessment, Development and Evaluation (GRADE) criteria. Then, this rating was downgraded if other three criteria were not met: high reporting quality according to shared standards, absence of internal methodological and statistical issues not detailed by the GRADE system, and absence of external validity issues.

**Results:**

With few exceptions, the GRADE rating, reporting and methodological quality of observational studies was “very low”, with frequent external validity issues. The only two randomized trials retrieved were, instead, of high quality. Only weak evidence was found for a relation between coagulopathy and mortality. Very weak evidence was found supporting the use of fibrinogen administration to reduce mortality in trauma. On the other hand, we found high evidence that the use of 1:1 vs. 1:2 high fresh frozen plasma/packed red blood cells ratios failed to obtain a 12% mortality reduction. This does not exclude lower mortality rates, which have not been investigated. The use of tranexamic acid in trauma was supported by “high” quality evidence according to the GRADE classification but was downgraded to “moderate” for external validity issues.

**Conclusions:**

Tranexamic acid is effective in reducing mortality in trauma. The other transfusion practices we investigated have been inadequately studied in the literature, as well as the independent association between mortality and coagulopathy measured with traditional laboratory testing. Overall, in this field of research literature quality is poor.

## Introduction

According to the WHO, trauma is one of the main causes of mortality worldwide with more than 1.2 million deaths per year, and the first among young people. [1]. To avoid death from hemorrhage in severely injured trauma patients, surgical or endovascular efforts to achieve hemostasis should be performed as early as possible. These treatments have been combined with transfusion algorithms supporting hemostasis. [2]. The attention of the scientific community has particularly focused on acute traumatic coagulopathy (ATC) which has frequently been recognized in severe trauma [3]. In different studies dealing with severe trauma using different and heterogeneous definitions of ATC, the prevalence of ATC ranges between 24 and 56% [4]. Treatment of coagulopathy is thought to reduce mortality in the severely bleeding trauma patients, and different therapeutic approaches have been proposed such as high ratios of fresh frozen plasma (FFP) and of platelets with packed red blood cells (PRBC), fibrinogen or cryoprecipitate administration and the use of antifibrinolytic drugs. However, there is still uncertainty on how to define coagulopathy and the efficacy of hemostatic algorithms [5, 6]. We therefore performed a systematic review and qualitative analysis based on strict methodological assessment of available literature, to verify the possibility of overoptimistic or unreliable results that have become a serious issue in medical literature [7, 8].

In February 2015 in the context of the 10^th^ edition of the Trauma Update and Organization Conference held in Bologna (Italy), a meeting of experts was organized with the goal of screening scientific evidence regarding trauma related coagulopathy, administration of high FFP:PRBC ratios, fibrinogen, and tranexamic acid, in severely injured patients. We report the results of the systematic review of the literature performed to support the works.

## Methods

The Coordinating Committee, formed by intensivists, surgeons, emergency department physicians, and experts in coagulation and transfusion practices generated the list of topics object of the review.

Four queries were formulated, one investigating the effects of coagulopathy in trauma and the other three focused on the effectiveness of different transfusion approaches. The four queries were formulated as follows:

1. Does coagulopathy affect mortality in trauma?
2. Does a fixed blood to plasma transfusion ratio reduce mortality in trauma?
3. Does hypofibrinogenemia treatment reduce mortality in trauma?
4. Does tranexamic acid administration reduce mortality in trauma?

### Literature Search Strategy

We searched the MEDLINE (using the PubMed platform) retrieving four lists of studies, one for each query, according to search strategies reported in the supporting information.

#### Study selection

Four groups of physicians, one for each query, were selected to screen the literature. Each group received the list of articles and performed the first selection on the basis of titles and abstracts excluding those that did not deal with the subject at hand. We selected only studies that included a study and a control group, including randomized controlled trials (RCTs) and observational studies only if adjustment for confounders was performed (e.g. logistic regression, matching). We also included meta-analyses and reviews for manual evaluation of the bibliography of the articles as a source of literature that may have escaped the PubMed search. We did not include letters, case reports, and observational studies without controls and adjustment for important covariates. Studies on children (age ≤ 16) were excluded. We excluded as well studies focusing on specific conditions such as head bleeding, considering instead abdominal, thoracic, pelvic bleeding, and coagulopathy in general. We excluded traumatic brain injury because of the great prognostic weight carried by even modest bleeding and because the outcome of choice in our review, mortality, is not suitable for traumatic brain injury studies for which a composite outcome including death, vegetative status, and severe disability is preferable. For generalizability issues we excluded studies carried out in the military setting. Such studies have provided fundamental evidence for therapeutic strategies in the field of trauma, but substantial differences in case-mix and health-care context hamper, in our opinion, the translation of evidence to the civilian context. Bleeding or coagulopathy from other causes (as obstetrical or perioperative) were not considered. Finally, we applied English-language restriction.

#### Data analyses

Full texts of the selected articles were collected and submitted for detailed methodological assessment by statistical experts (DP, AC).

Data extraction was performed according to a predefined plan (the protocol was not pre-registered), using a dedicated electronic form that automatically performed all planed computations and plot generation already tested in a previous reviewing process performed by the Trauma Update review group [9].

We presented data as event rates in treatment arms and controls, absolute risks, absolute risk reductions, and relative risks. We reported multivariate analysis results as adjusted odds or hazard ratios. Numbers needed to treat were also calculated when appropriate. We calculated confidence intervals for all the above measures. Confidence intervals for the number needed to treat include the area of numbers to treat for benefit (NNTB) and the area of numbers needed to treat to be harmed (NNTH), separated by an infinity value which corresponds to an absolute risk difference of zero [10]. We represented absolute and relative risks from RCTs in Forest plots.

#### Evidence grading

We ranked the evidence provided by RCTs and observational studies according to the Grading of Recommendations Assessment, Development and Evaluation (GRADE) criteria without providing any recommendation because it was not an objective of this study. GRADE rates evidence quality on a four-level scale ranging from “high” to “very low”, according to confidence that raters have on the estimates of the effect [11]. RCTs provide default “high” quality that can be downgraded if bias or other limitations are present. Observational studies, instead, are initially rated as “low” quality but can be up- or downgraded depending on specific features. Consistently with shared evidence, the GRADE rating system considers crucial adequate control for confounding which implies that when a models purpose is explanatory, at least most known prognostic factors should be measured and included in the model [12–14].

Although GRADE rating has been developed for bodies of evidence, we applied its evidence quality criteria first to single studies and proceeded with body evidence analysis only in a further step. According to GRADE rating, “very low” quality indicates that the degree of the estimate uncertainty of the documented effects is so high as to be compatible with substantially different true effects (including absence of any effect).

We provided a further quality assessment based on statistics reporting (which was graded as “partial” or “sufficient for quality assessment”) and methodological/statistical quality (“low” or “high”). Finally, we verified the existence of external validity issues (“yes” or “no”).

We combined studies in meta-analyses and used statistical approaches to investigate presence of asymmetry and potential publication only if heterogeneity did not advise against these procedures [15].

In synthesis, we rated evidence quality according to GRADE only after having verified that the studies complied with three additional criteria:

1. High quality reporting (rated “partial” or “sufficient for quality assessment”):
  - According to the Consolidated Standards of Reporting Trials (CONSORT) statement for RCTs [16].
  - According to indications provided by literature for observational studies [17].
2. Absence of methodological and statistical flaws. Specifically:
  - Flaws in regard to RCTs, defined by the CONSORT recommendations.
  - Flaws which can affect observational studies, not detailed by the GRADE system such as:
    Risk of overfitting when less than 10 outcomes per variable are available [18, 19]; bivariate statistics tests used to screen variables for multivariable analysis [20]; abuse of automatic variable selection procedures [21]; not accounting for immortal-time bias when dealing with time-dependent treatments [22]; not balancing the probability of receiving a specific treatment with propensity scores [23].
3. Absence of external validity issues, such as specificity of case-mix, of treatment protocols, of health-care settings [5].

If these criteria were not met we downgraded the GRADE rating.

The review was conducted complying with the Preferred Reporting Items for Systematic Reviews and Meta-Analyses (PRISMA) statement recommendations (S1 PRISMA Checklist) [24].

## Results

### Query # 1: Does coagulopathy affect mortality in trauma?

**Fig 1:**
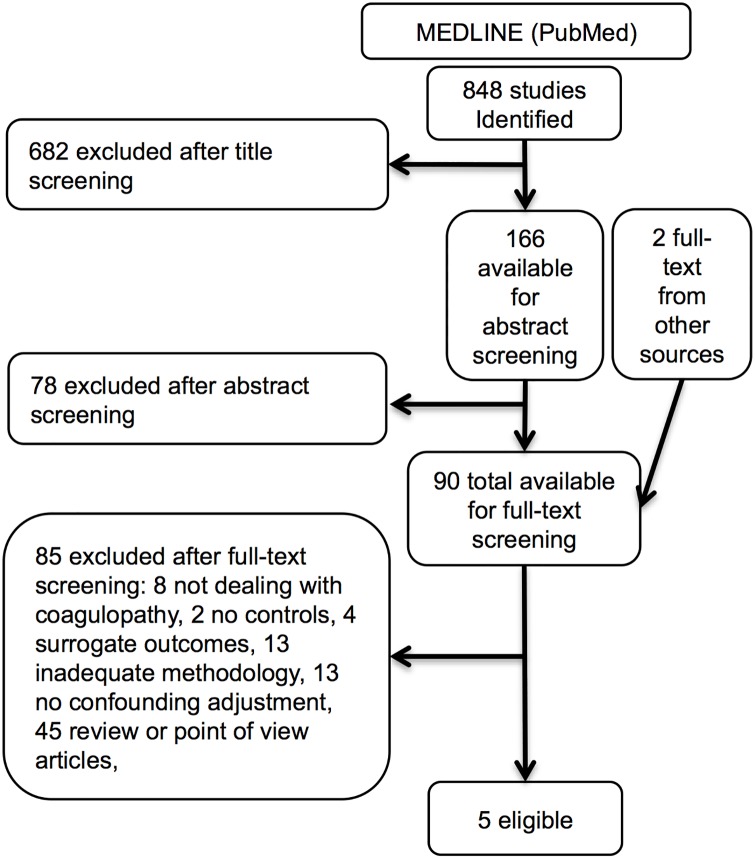
Query # 1: Does coagulopathy affect mortality in trauma? Studies selection flow diagram.

Out of 848 studies five were selected after application of exclusion criteria (Fig 1, Table 1, and S1 File) [25–29]. Definition of coagulopathy was heterogeneous among studies: hypofibrinogenemia, platelet reduction, increased activated partial thromboplastin time (APTT), prothrombin time (PT), or international normalized ratio (INR). Inclusion criteria and case-mixes were also different ranging from all patients with trauma [25] to those with trauma receiving full trauma team activation (without any further specification) [26], to patients receiving different number of packed red blood cells (PRBC) over different timespans [27, 28], to those with low systolic blood pressure and not better specified “poor responsiveness to initial fluid resuscitation” [29].

**Table 1:** Query # 1: Does coagulopathy affect mortality in trauma? Reporting of studies included in the revision. The number of patients is referred to those included in the multivariable model. N = number, pts = patients, ctr = centre, OR = odds ratio, CI = confidence interval, APTT = activated partial thromboplastin time, PT = prothrombin time, INR = international normalized ratio.

First author—Year	N of pts	N of centres	pts/ctr/year	Inclusion criteria	Coagulopathy indicated by	Outcome Mortality N (%)	Mortality OR (95%-CI)
Hagemo– 2014 [26]	1133	4	142	Patients initiating full trauma team response, with time from injury to arrival within 180 minutes	Fibrinogen and platelet reduction, INR increase	28-day mortality: 99 (8.7)	Low fibrinogen 0.08 (0.03–0.20). High fibrinogen 1.77 (0.94–3.32). INR 1.65 (0.65–4.18). Platelet count 1 (1.0–1.0).
Rourke– 2012 [29]	517	2	86	Patients who met criteria for local trauma team activation, time from injury to arrival within 120 minutes, less than 2000 ml fluid administration prior to hospital arrive	Fibrinogen reduction, APTT increase	28-day mortality: 62 (12)	Fibrinogen 0.22 (0.10–0.47). APTT 1.05 (1.01–1.09).
Mitra– 2010 [27]	331	1	90	Patients receiving more than 4 packed red blood cell units within 4 hours from admission	INR increase, Platelet count reduction	30-day mortality: 99 (29.9)	Platelet count 0.99 (0.99–0.99). INR 1.43 (1.02–2.01).
MacLeod– 2003 [25]	7638	1	1272	All patients with trauma	PT and APTT increase	Hospital mortality: NA	PT 1.35 (1.11–1.68). APTT 4.26 (3.23–5.62).
Sambavisan– 2011 [28]	1181	23	22	Patients receiving at least one but less than 10 PRBC units within 24 hours from admission	APTT increase	Hospital mortality: 173 (14.6)	APTT 1.015 (1.010–1.019).

In four studies statistical reporting concerning multivariate analysis was inadequate (S1 File) [25, 27–29]. One of these studies dichotomized all the variables included in the model without adequately justifying this approach and the cut-offs chosen [25]. Only one study had fair reporting of statistics that appeared to be correctly performed [26]. This study, however, provided a biphasic, paradoxical, independent effect of fibrinogen increase on mortality. It turned out to strongly reduce mortality within low plasmatic levels, and to increase it at high concentrations [26]. A similar paradoxical effect was observed for increasing values of the injury severity score (ISS), with increased risk of death below the breakpoint of 25.7 and reduced mortality for higher values [26]. This raises serious concerns on the adequacy of the modeling process.

Three studies included only five variables in the mortality prediction models [27–29] and important well-known prognostic factors were ignored such as age (omitted in one of the two models developed in one study [28]), severity on admission (omitted in one study) [27], or Glasgow coma scale (GCS) not included in two models [28, 29].

Another issue was result’s generalizability since only one study included a high number of centers and patients (1,181) [28], a second one included four major trauma centers from three different countries (1,133 patients included in the model) [26], a third one was carried out only in two centers (517 patients) [29], and two were single center studies (7,638 and 331 patients, respectively) [25, 27].

Conclusion: Because of heterogeneity in design and definition of coagulopathy, evidence from different studies could not be combined as recommended by the GRADE working group. Each single study provided “very low” evidence according to the GRADE rating system.

Statistical reporting was partial and the assessable statistical analyses were of “low” quality. Only one study did not raise generalizability concerns.[28]

We, therefore, provided a conclusive “very low” rating to evidence quality.

### Query # 2: Does a fixed blood-plasma transfusion ratio reduce mortality in trauma?

**Fig 2:**
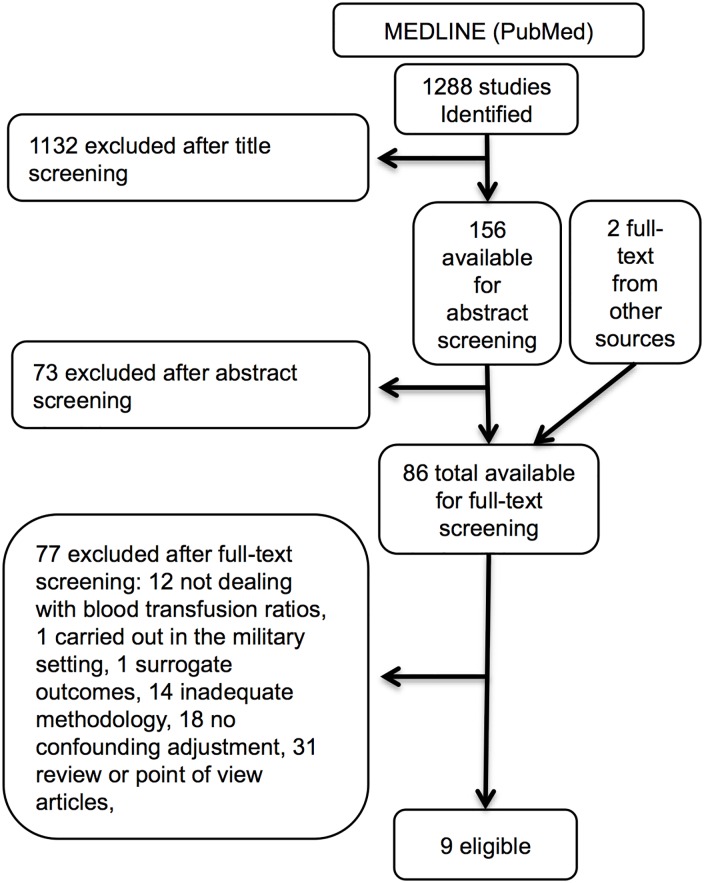
Query # 2: Does a fixed blood-plasma transfusion ratio reduce mortality in trauma? Studies selection flow diagram.

We identified 1,288 studies, screened twenty-five full-texts, and after applying our exclusion criteria we reviewed nine (Fig 2, Table 2, and S2 File) [27, 28, 30–36]. These nine studies were quite heterogeneous in terms of treatment protocols, comparing several FFP/PRBC ratios, or instead considering FFP/PRBC ratios as a continuous variable in multivariate analysis, or comparing patients that received or not FFP. Inclusion criteria were also quite different with one study dealing only with ICU patients [31], one selecting patients on the basis of a severity score value [33], two studies focusing on patients transfused with 10 or more PRBC within 24 hours from admission [34, 35], and five based on other PRBC transfusion thresholds [27, 28, 30, 32, 36]. Study heterogeneity in terms of case-mix, treatment protocols, and outcome advised against combination for overall evidence provision.

**Table 2:** Query # 2: Does a fixed blood-plasma transfusion ratio reduce mortality in trauma? Reporting of observational studies included in the revision. The number of patients is referred to those included in the multivariable model. N = number, pts = patients, ctr = centre, FFP = fresh frozen plasma, PRBC = packed red blood cells, OR = odds ratio, HR = hazard ratio, CI = confidence interval, Cont. Var. indicates that variables are used as continuous in the models, when not specified they have been categorized.

First author—Year	N of pts	N of centres	pts/ctr/year	Inclusion criteria	Outcome	Mortality (%)	Mortality OR (95%-CI)*
Scalea– 2008 [31]	NA	1	NA	Patients admitted to the ICU for trauma occurred within 24 hours	Hospital mortality	NA	PRBC:FFP ratio 1:1 0.57 (0.19–1.66). PRBC:FFP ratio (Cont. Var.) 1.23 (0.81–1.87)
Inaba– 2010 [36]	568	1	95	Trauma admitted to surgical ICU receiving < 10 PRBC units within 12 hours from admission (excluding deaths occurred within 24 hours)	Hospital mortality	89 (15.7)	FFP 1.27 (0.81–2.0)
Wafaisade– 2011 [32]	1362	116	3	Patients survived one hour from admission receiving more than 3 and less than 10 PRBC units from arrival to the ER and admission to the ICU	Hospital mortality	321 (23.6)	FFP:PRBC ratio <1:1 reference. FFP:PRBC ratio = 1:1 0.8 (0.54–1.18) FFP:PRBC ratio >1:1 0.52 (0.31–0.87)
Holcomb– 2013 [30]	876	10	79	Trauma patients receiving at least 3 PRBC units within 24 hours from admission	Hospital mortality	NA	FFP:PRBC ratio > = 1:1 HR 0.23 (95%-CI NA) FFP:PRBC ratio: ≥ 1:2—<1:1 HR 0.42 (95%-CI NA) FFP:PRBC ratio < 1:2 HR ref = 1 (95%-CI NA) FFP:PRBC (Cont. Var.) HR 0.31 (0.16–0.58)
Teixeira– 2009 [35]	383	1	64	Trauma patients receiving 10 or more PRBC units within the first 24 hours	Hospital mortality	161 (42)	FFP:PRBC ratio 0.02 (0.01–0.07)
Sambavisan– 2011 [28]	1181	23	22	Patients receiving at least one but less than 10 PRBC units within 24 hours from admission (excluding patients dies within 2 hours from admission)	Hospital mortality	173 (14.6)	FFP:PRBC ratio ≥1 HR 0.87 (0.55–1.38)
Holcomb– 2011 [34]	643	22	29	Trauma patients receiving 10 or more PRBC units within 24 hours from admission	30-day mortality	181 (28.1)	FFP:PRBC ratio (Cont. Var.) HR 0.49 (0.28–0.86)
Borgman– 2011 [33]	557	100	1	TASH score ≥ 15 excluding patients died within 1 hour from admission	Hospital mortality	NA	FFP:PRBC ratio (Cont. Var.) Survival OR 2.5 (1.56–4.00)§
Mitra– 2010 [27]	331	1	90	Patients receiving more than 4 packed red blood cell units within 4 hours from admission	30-day mortality	99 (29.9)	FFP:PRBC ratio (Cont. Var.) 0.15 (0.05–0.48)

* When the chosen multivariable analysis is a proportional-hazards regression model, the result is preceded by the acronym “HR”, in all the other cases ORs from logistic regression are implied.

^§^ In this case the survival and not the mortality OR was calculated.

All but one study [36] did not develop a propensity score to account for the selection bias associated with therapeutic choices.

Two studies adhering to the Trauma Registry-Deutsche Gesellschaft für Unfallchirurgie included about one hundred centers but, on average, only few patients per center, raising a selection bias issue [32, 33]. On the other hand results from the four single center studies reasonably may have had limited external validity [27, 31, 35, 36].

In general the statistical reporting was scanty and inadequate for quality evaluation. Impact of FFP administration was also variable, with only six of eight studies showing a survival benefit.[30, 32–35]

Compared to the others, the Prospective, Observational, Multicenter, Major Trauma Transfusion (PROMMTT) study was of higher statistical quality since it accounted for immortal-time bias and reported adequately the statistical design and results.[30] However, it was not upgraded since in the face of a large effect it was probably underfitted for its explanatory purpose and did not include a propensity score. Thus, it provided “low evidence” according to GRADE, with adequate statistical reporting and quality, and no external validity issues.

The study reported a large and statistically significant mortality reduction in the first six hours from admission, but no protective effect of high FFP/PRBC ratios (categorized on three levels: < 1:2 as the reference, vs. ≥ 1:2—< 1:1, and vs. ≥ 1:1) between 6 and 24 hours, and 24 hours and 30 days.

Although, published after the conclusion of our literature search, we included in our analysis a recent randomized controlled trial (RCT), the Pragmatic, Randomized Optimal Platelet and Plasma Ratios (PROPPR) trial (Table 3) [37]. This study recruited 680 severely injured patients admitted to 12 level I trauma centers in the United States, randomizing patients to a FFP/platelet/PRBC 1:1:1 vs. 1:1:2 ratio. No statistically significant 28-hours mortality (12.8 and 17%, respectively) or 30-day mortality reduction was found (22.4 and 26.1%, respectively). The reporting and the methodology were adequate, so we gave a “high” quality rating according to the GRADE criteria (S2 File).

**Table 3:** Query # 2: Does a fixed blood-plasma transfusion ratio reduce mortality in trauma? Results of the Pragmatic, Randomized Optimal Platelet and Plasma Ratios (PROPPR) trial [37]: 24-hour and 30-day mortality are reported. RR = relative risk, CI = confidence interval, NNTB = number needed to treat for benefit, NNTH = number needed to treat to be harmed, FFP = fresh frozen plasma, PRBC = packed red blood cells.

Treatment	Control	Investigated Outcome	% (95%-CI) Treatment arm	% (95%-CI) Controls	% (95%-CI) Difference	RR (95%-CI)	NNTB/NNTH
FFP/platelet/PRBC Ratio 1:1:1–335 patients	FFP/platelet/PRBC 1:1:2–341 patients	24-hours mortality	12.8 (9.7 to 16.8)	17 (13.4 to 21.4)	-4.2 (-9.6 to 1.2)	0.75 (0.52 to 1.09)	NNTB 24 (95%-CI NNTB 10 to ∞ to NNTH 82)
FFP/platelet/PRBC 1:1:1–335 patients	FFP/platelet/PRBC 1:1:2–341 patients	30-day mortality	22.4 (18.3 to 27.2)	26.1 (21.7 to 31)	-3.7 (-10.1 to 2.8)	0.86 (0.66 to 1.12)	NNTB 27 (95%-CI NNTB 10 to ∞ to NNTH 36)

The high-quality observational study [30] and the RCT [37] provided results that partially converged on a common body of evidence. Both studies were carried out in level-1 trauma centers and included patients for whom the highest level of activation was required. Moreover, high FFP/PRBC ratios 1:1 (or higher in the observational study) were compared with ratios 1:2 (or lower in the observational studies). Finally, overall 24-hour mortalities were similar in the two groups (13.0% in the observational study and 14.8% in the RCT).

The observational study did not report an adjusted analysis based on cumulative mortality, but indicated a very early (i.e. first six hour from admission) protective effect of high ratios, and no protective effect in the 6-hour to 24-hour and 24-hour to 30-day models. The RCT considered instead cumulative 24-hour and 30-day mortalities. These results suggest that even if a very early effect may be present (“low evidence” provided by the observational study) in the medium and long period no beneficial effect is detected (“high evidence” mainly attributable to the RCT).

Conclusion: Of the nine selected observational studies eight were downgraded to “very low” according to the GRADE rating system. Further, they provided “partial” statistical reporting and had “low” methodological quality, most having external validity limits. Our final evaluation confirmed the “very low” GRADE rating.

We confirmed the “low” GRADE rating also for the PROMMTT study for which we did not have generalizability, statistical reporting and quality concerns [30]. The PROPPR trial was a well-conducted RCT with “high” evidence quality according to GRADE and confirmed in our final evaluation [37].

The two studies were sufficiently homogeneous to provide cumulative “high” level evidence against the greater efficacy of 1:1 vs. 1:2 FFP/RPBC ratios.

### Query # 3: Does hypofibrinogenemia treatment reduce mortality in trauma?

**Fig 3:**
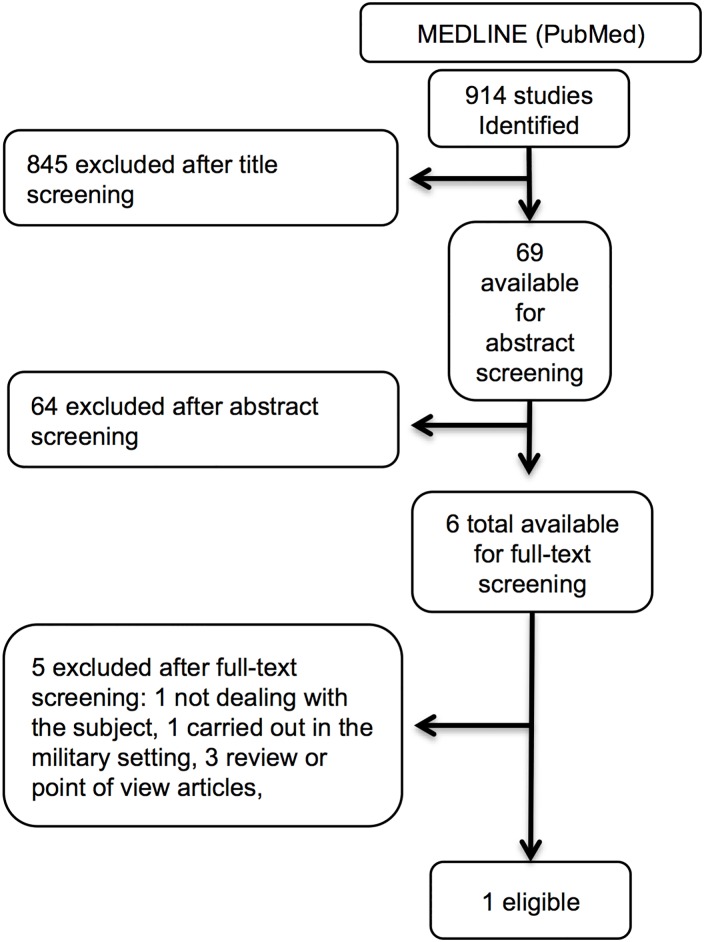
Query # 3: Does hypofibrinogenemia treatment reduce mortality in trauma? Studies selection flow diagram.

Of the 914 retrieved studies five were left for full-text screening, but only one matched our inclusion criteria (Fig 3, Table 4, and S3 File) [29]. The study included patients with suspected active hemorrhage, hypotension, and scarce response to fluid resuscitation (S3 File, Table 4). Individual fibrinogen doses were calculated on the basis of FFP, platelet, and cryoprecipitate that were administered. In the 28-day logistic regression model fibrinogen administration within 12 hours (conditional on 12-hour survival) seemed to have a protective effect, the result being barely not significant (OR 0.91; 95%-CI 0.81–1.01). The statistical reporting was however too poor for a correct evaluation. The model included only five variables, did not include a propensity score for fibrinogen administration, and did not account for immortal-time bias.

**Table 4:** Query # 3: Does hypofibrinogenemia treatment reduce mortality in trauma? Reporting of studies included in the revision. The number of patients is referred to those included in the multivariable model. N = number, pts = patients, ctr = centre, OR = odds ratio, CI = confidence interval.

First author—Year	N of pts	N of centres	pts/ctr/year	Inclusion criteria	Outcome Mortality (%)	Treatment	OR (95%-CI)
Rourke– 2012 [29]	517	2	86	Time from injury to arrival within 120 minutes, SBP < 90 at admission, poor responsiveness to initial fluid administration	28-day mortality 62 (12)	Fibrinogen administration within the first 12 hours	Fibrinogen 0.91 (0.81–1.01).

Conclusion: Evidence from this study was rated “very low” according to the GRADE classification, statistical reporting was “partial” and quality of reported statistics was “low”, we raised generalizability issues since only two centers participated to the study. Our final judgment confirmed the “very low” GRADE category.

### Query # 4: Does tranexamic acid administration reduce mortality in trauma?

**Fig 4:**
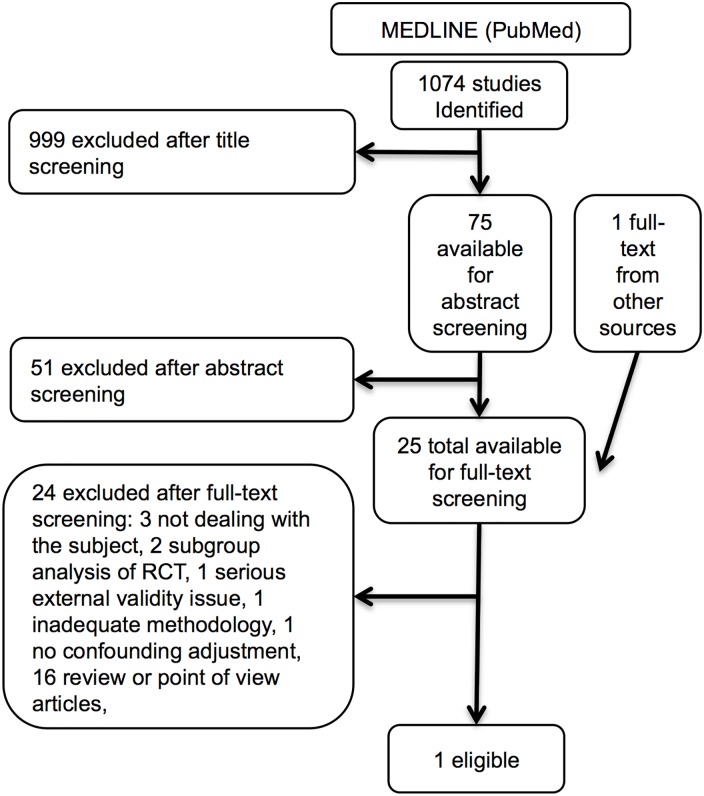
Query # 4: Does tranexamic acid administration reduce mortality in trauma? Studies selection flow diagram.

The PubMed search provided 1,074 results, 25 studies were fitted the criteria for full-text screening and 24 were discarded (Fig 4, Table 5, and S4 File). Only the 2010 CRASH-2 trial was left for evidence provision [38]. This was a very large, multicenter, international trial including trauma patients with systolic blood pressure lower than 90 mmHg and/or heart rate higher than 110 beats per minute or who were considered at risk of significant hemorrhage. Patients were randomized to receive either tranexamic acid or placebo within 8 hours from injury. The reporting of statistics, design, and results was adequate and we rated study quality as “high” according to the GRADE classification. Statistically significant 28-day mortality reduction was found with a relative risk of 0.91 (95%-CI 0.85–0.97; *p* = 0.0035). Absolute mortality reduction was 1.5% (95%-CI 0.5–2.5) from 16% for patients receiving placebo to 14.5% in the treatment group, the number needed to treat for benefit being 68 (95%-CI 40–206).

**Table 5:** Query # 4: Does tranexamic acid administration reduce mortality in trauma? Results of the CRASH 2 trial [38]. The number of patients is referred to those included in the multivariable model. CI = confidence interval, RR = relative risk, NNTB = number needed to treat for benefit.

Treatment	Control	Investigated Outcome	Mortality rate in the treatment arm % (95%-CI)	Mortality rate in controls % (95%-CI)	% (95%-CI) Difference	RR (95%-CI)	NNTB/NNTH
Tranexamic acid—10060 trauma patients	Placebo—10067 trauma patients	28-day mortality	14.5 (13.9 to 15.2)	16 (15.3 to 16.8)	-1.5 (-2.5 to -0.5)	0.91 (0.85 to 0.97)	NNTB 68 (95%-CI NNTB 40 to NNTB 206)

Conclusion: Evidence provided by the study was rated “high” according to the GRADE classification, statistical reporting was adequate, and statistical quality was high. However, the study was carried out mainly in developing countries raising an issue of generalizability to Western countries health-care contexts. We thus downgraded to “moderate” the quality of evidence in our final evaluation.

## Discussion

Although a great number of articles dealing with coagulopathy and transfusion practices in trauma have been published, only few in our revision were rated as having an acceptable quality to be a reliable source of evidence. Moreover, heterogeneity in design, definitions, and treatment protocols was high, hampering the combination of single study results in an unique body of evidence, as recommended by the GRADE.

During the revision process, besides insufficient statistical reporting, we frequently encountered internal validity issues connected to the inappropriate use of multivariable models. Besides technical flaws, such as low variable/outcome ratios or bivariate use for variables selection consistent with similar findings in other fields of research [17, 39–41], models were frequently underfitted for their explanatory purposes [14]. This means that the number of important predictors included in the models was insufficient to prove that the association between variables (e.g. a specific treatment) and outcome was causal in nature, because of the high risk of confounding. Underfitted models may exaggerate the quantitative relations between specific variables and outcomes or even present spurious associations as causal.

Another weak point of most studies, was the small number of centers participating (many studies were carried out in one or two centers), which may raise generalizability issues when study centers have specific case-mix and health-care organization features [5].

Crucial for the aim of our study (i.e. grading the evidence in support of transfusion practices in trauma, finalized at improving survival) was to define coagulopathy and assess its relation with standardized mortality as a prerequisite for understanding the potentials for treatment. Literature review showed lack of agreement with regard to the definition of acute trauma coagulopathy, which was heterogeneous across selected studies, though mainly based on hypofibrinogenemia or prolongation of PT or APTT, or INR increase over arbitrary cut-offs. Unfortunately, the studies we scrutinized that investigated the independent association between coagulopathy and death included few variables in their multivariable models, often omitting important predictors, an inadequate approach when the purpose is explanatory (i.e. the correct characterization of the relationship between independent variables and the outcome) and not prognostic [14]. Although, a relation between coagulopathy and death is plausible and does not need any demonstration, to target specific treatments we should be able to discriminate between patients that are dying because they are bleeding and those that are bleeding as a terminal event of severe trauma.

Interestingly, the studies dealing with FFP/PRBC ratios adopted very heterogeneous inclusion criteria, witnessing, in our opinion, the difficulty of researchers in defining the target population for treatment, generically focusing on patients with severe bleeding which includes a large range of definitions. Severity was often not defined by clinical conditions but by therapy, i.e. a high number of PRBC administered in a certain time frame, for example 24 hours. This definition, however, generates a paradox because when translated into clinical practice it requires the prediction on hospital admission of the amount of PRBC units a patient will receive in the following hours to select those for whom the high FFP/PRBC ratio is indicated. Physicians may discriminate reliably between extreme conditions, i.e. patients with very severe bleeding on one hand and those with limited severity on the other, but probably not within intermediate degrees of severity. Patients at high risk of dying for coagulopathy should be instead spotted using variables collectable at admission. To develop an accurate predictive model, however, permanent registries involving tenths of centers and thousands of patients are needed, and a high number of variables for each patient need to be collected. Unfortunately, the achievement of this goal seems currently out of sight.

Among the studies the PROMMTT had a high statistical quality, accounting for the immortal-time bias [30]. This bias is attributable to the higher chance of receiving FFP transfusion that patients who survive the first few hours after admission have. Thus, survival becomes the condition to receive treatment and not the opposite. Treatment is thus a time-dependent variable that should be modeled properly when using multivariable approaches. Modeling should account for the fact that the timeframe between admission and treatment is a period of survival (i.e. the immortal time) without exposure to treatment [22]. Unfortunately, this study did not account for selection bias with a propensity score and was probably underfitted to account adequately for confounding. This study analyzed separately the adjusted effect of high FFP/PRBC ratios on mortality in the first 6 hours from admission, between 6 and 24 hours, and between 24 hours and 30 days. Treatment was effective only in the earliest period. However, early mortality is not the best outcome when dealing with severely ill patients because it does not account for late mortality related to the initial injury. This approach will not recognize potential initial “cosmetic” effects which may avoid early deaths, but actually only delay the outcome.

Since there is no analysis focused on cumulative delayed mortality in the PROMMTT study, its results should not be compared with those from the PROPPR trial that reported no statistically significant 24-hours and 30-day mortality reduction attributable to administration of 1:1 vs. 1:2 FFP/PRBC ratios. The RCT provides “high” evidence against a large protective effect (12% mortality reduction assumed for sample size calculation in the RCT) of high FFP/PRBC ratios. Such a large protective effect was probably an excessively ambitious goal of the PROPPR study, not thoroughly compliant with the principle that the minimal clinically relevant difference (say, a 5% mortality reductions would have been a more than fair objective) should guide sample size computation [42]. This means that though there is high evidence against the use of 1:1 ratios to obtain a 12% mortality reduction, there is no evidence that, say, a 5% mortality reduction could or could not be achieved, and “absence of evidence is not evidence of absence” [43].

Our results are in contrast with the evidence quality assessment provided by two recent guidelines [44, 45]. Hunt et al. rated as “moderate” evidence quality (B grade according to the GRADE classification) concerning 1:1 FFP:PRBC ratios in trauma, on the basis of achieved hemostasis and exsanguination results from the PROPPR trial [44]. Unfortunately, the assessors of these subjective endpoints were not blinded with regard to treatment assignment at that point of the study, with a high risk of detection bias [5]. Mainly for the same reason Rossaint et al. also rated as “moderate”, evidence in favor of FFP:PRBC ratios ≥ 1:2 for the treatment of severely bleeding trauma patients [45]. The rating attributed to evidence in support of high FFP:PRBC ratios was excessively optimistic in our opinion, since the PROPPR trial failed to demonstrate any survival advantage (the primary outcome of the study) and the remaining literature mostly provided insufficient evidence.

We only selected one study dealing with tranexamic acid administration, which was the only RCT performed in this field [38]. The study was correctly designed and performed, and was provided with a high evidence level. We however have doubts on its generalizability to Western countries, since most patients were enrolled in developing countries with very different health-care systems. We hypothesized, for example, that time from trauma to surgery could be prolonged in some of these countries. In this case the contrast of fibrinolysis could prolong survival time increasing the chance of receiving surgical hemostasis. This beneficial effect could be less relevant (the study demonstrated a 1.5% mortality reduction) when surgical hemostasis is timely. In the CRASH 2 trial about half the sample did not receive any blood product. It is likely that a minority of patients died early preventing any transfusion treatment but that most were at very low risk of death. The use of broad inclusion criteria in this study may have diluted the results of the study, which showed only a 1.5% 28-day mortality reduction. We may reasonably hypothesize that a significantly greater impact would have observed in cases severe hemorrhage.

Finally, no evidence was found in support of fibrinogen administration in the bleeding traumatic patient, since the only selected study bore paradoxical results [29]. Moreover, fibrinogen was administered as FFP, platelets, and cryoprecipitate. FFP contains other coagulation factors besides fibrinogen and requires high volume administration determining hemodilution. Thus, it is probably not the best choice when the objective is to compensate fibrinogen consumption. In this case too our evaluation was discordant with that of recent European guidelines [45], which attribute a C grade level of evidence, basing their evaluation of fibrinogen administration efficacy on a single study carried out in a combat setting, which did not account for the immortal-time bias, did not use a propensity score, and adopted a clearly underfitted model with only three variables [46].

On the other hand, the use of fibrinogen in clinical practice which is spreading in cases of massive hemorrhages is based on solid theoretical basis, and, therefore, should not be abandoned solely because it has been inadequately studied, again consistently with the principle that “absence of evidence is not evidence of absence” [43].

### Study limitations

The main limit of our review was that, due to limited resources, we only searched the MEDLINE database using the free PubMed provider. We also applied very strict methodological criteria, which made our analysis sensitive but may have ended up excluding articles potentially valuable for the study of coagulopathy in trauma.

### Conclusions

Tranexamic acid is effective in reducing mortality in trauma. High 1:1 FFP/PRBC ratios are not effective in determining a 12% mortality reduction compared to 1:2 ratios. Fibrinogen administration in trauma has not been studied adequately, and its effectiveness, supported by a strong theoretical rational, cannot be excluded. The definition and prognostic role of traumatic coagulopathy has been insufficiently addressed by current literature.

The difficulty to perform trials in the field is witnessed by the fact that in our review we retrieved only two RCTs. A large amount of observational studies, instead, have been carried out in this field but only few were of sufficient quality. Nevertheless, observational studies still appear to be the most feasible solution for evidence gathering in this field. Our suggestions for future observational studies are summarized in S5 File.

## Supporting Information

S1 File MEDLINE database search, flow diagram illustrating the literature selection process, and evidence assessment for the first query. (PDF)

S2 File MEDLINE database search, flow diagram illustrating the literature selection process, and evidence assessment for the first query, and results from the PROPPR trial. (PDF)

S3 File MEDLINE database search, flow diagram illustrating the literature selection process, and evidence assessment for the first query. (PDF)

S4 File MEDLINE database search, flow diagram illustrating the literature selection process, evidence assessment for the first query, and results from the CRASH2 trial. (PDF)

S5 File Suggestions for future observational studies. (PDF)

S1 PRISMA Checklist In this file we report the Checklist of items included in our systematic review according to the Preferred Reporting Items for Systematic Reviews and Meta-Analyses (PRISMA) statement recommendations. (PDF)
