# Supplementary material for: Blood Component Therapy and Coagulopathy in Trauma: A Systematic Review of the Literature from the Trauma Update Group
Source: PLoS One. 2016 Oct 3;11(10):e0164090. doi: 10.1371/journal.pone.0164090 (PMC5047588; doi:10.1371/journal.pone.0164090)
Supplement: S1 File — (PDF) [file pone.0164090.s001.pdf]

S1-file: MEDLINE database search, flow diagram illustrating the literature selection process, and evidence assessment for the first query.

Query # 1: *"Does coagulopathy affect mortality in trauma?".*

PubMed search details: search date December 9 2014

((("blood coagulation disorders"[MeSH Terms] OR ("blood"[All Fields] AND "coagulation"[All Fields] AND "disorders"[All Fields]) OR "blood coagulation disorders"[All Fields] OR "coagulopathy"[All Fields]) AND ("injuries"[Subheading] OR "injuries"[All Fields] OR "trauma"[All Fields] OR "wounds and injuries"[MeSH Terms] OR ("wounds"[All Fields] AND "injuries"[All Fields]) OR "wounds and injuries"[All Fields])) AND ("mortality"[Subheading] OR "mortality"[All Fields] OR "mortality"[MeSH Terms])) AND ("2000/01/01"[PDAT]: "3000"[PDAT])

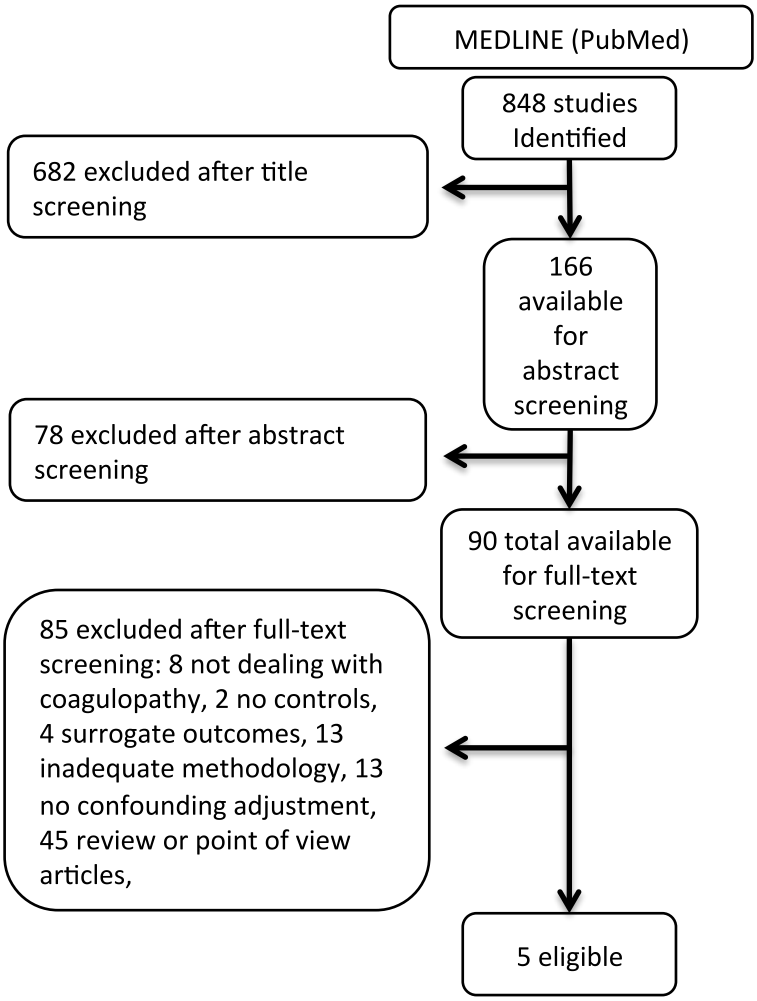
MEDLINE (PubMed)
848 studies Identified
682 excluded after title screening
166 available for abstract screening
78 excluded after abstract screening
90 total available for full-text screening
85 excluded after full-text screening: 8 not dealing with coagulopathy, 2 no controls, 4 surrogate outcomes, 13 inadequate methodology, 13 no confounding adjustment, 45 review or point of view articles,
5 eligible

Table S1

Observational study	1	Very low evidence - Downgraded study
**Year**	2014	
**Journal**	CC	
**First Author**	Hagemo	
**Statistical method**	Generalised additive regression and piecewise linear model	
**Inclusion criteria**	Patients initiating full trauma team response with time from injury to arrival within 180 minutes	
**Risk factor**	Fibrinogen and platelet reduction, INR increase	
		**Outcome**
		28-day mortality: NA
**Centres**	4	**Variable: OR (95%-CI)**
**N° patients/centre/year**	NA	Low fibrinogen: 0.08 (0.03-0.20)
**Study duration (days)**	729	High fibrinogen: 1.77 (0.94-3.32)
**Total (included in the model)**	1133	INR: 1.65 (0.65-4.18)
		Platelet count: 1 (1.0-1.0)
	**GRADE CRITERIA**	
	**Statistical reporting**	Partial
	**Methodological and statistical**	Low
**Downgrading**	Appropriate eligibility criteria	Yes
	Measurement of exposure	Yes
	Measurement of outcome	Yes
	Adequate control for confounding	No
	Bias	very serious
**Up-grading**	Size of effect	Very large
	Residual confounding	Does not indicate upgrading
	Dose /response	Yes
	**DETAILS**	
**Downgrading**		
**Up-grading**	Dose /response: FFP:PRBC ratio significant as a continuous variable generated, however, by a probably biased model. Upgrading not indicated.	
**External validity**	Only two centres participating to the study, with, however, a high number of patients treated per centre	
**Conclusive evaluation**	GRADE rating up/down	Downgraded study
	GRADE rating	Very low evidence
	Statistical reporting	Partial
	Methodological and statistical quality	Low
	External validity issues	Yes
	Final grading	Downgraded study
	Final level of evidence	Very low evidence

Table S1 (continued from the previous page)

Observational study	2	Very low evidence - Downgraded study
**Year**	2012	
**Journal**	JTH	
**First Author**	Rourke	
**Statistical method**	Logistic regression	
**Inclusion criteria**	Time fron injury to arrival within 120 minutes, SBP < 90 at admission, poor responsiveness to initial fluid administration	
**Risk factor**	Fibrinogen reduction, APTT increase	
		**Outcome**
		28-day mortality: 62 (12%)
**Centres**	2	**Variable: OR (95%-CI)**
**N° patients/centre/year**	86	Fibrinogen: 0.22 (0.10-0.47)
**Study duration (days)**	1095	APTT: 1.05 (1.01-1.09)
**Total (included in the model)**	517	
**28-day mortality**	62 (12%)	
	**GRADE CRITERIA**	
	**Statistical reporting**	Partial
	**Statistical quality**	Low
**Downgrading**	Appropriate eligibility criteria	Yes
	Measurement of exposure	Yes
	Measurement of outcome	Yes
	Adequate control for confounding	No
	Bias	very serious
**Up-grading**	Size of effect	Large
	Residual confounding	Does not indicate upgrading
	Dose /response	Not applicable
	**DETAILS**	
**Downgrading**	Adequate control for confounding: Only four variables entered the final model, too few for explanatory purposes. Statistical reporting : Logistic regression. No Statistical support reported. Checking for conformity with linear gradient for continuos variables not reported. Test for interaction not reported. Goodness-of-fit assessment not reported. Collinearity assessment not reported. Statistical tests for models not reported. Variable selection method: Stepwise forward selection. Reporting of variable coding method not performed. Methodological and statistical quality: Insufficient statistical reporting. Important covariates are missing in the model (possible underfitting).	
**Up-grading**	Size of effect: The large fibrinogen OR reduction may be the result of a biased model. Upgrading is not indicated.	
**External validity**	Only four centres participating to the study, with, however, a high number of patients treated per centre	
**Conclusive evaluation**	GRADE rating up/down	Downgraded study
	GRADE rating	Very low evidence
	Statistical reporting	Partial
	Methodological and statistical quality	Low
	External validity issues	Yes
	Final grading	Downgraded study
	Final level of evidence	Very low evidence

Table S1 (continued from the previous page)

Observational	3	Very low evidence - Downgraded study
**Year**	2010	
**Journal**	Injury	
**First Author**	Mitra	
**Statistical method**	Logistic regression	
**Inclusion criteria**	Patients receiving more than 4 packed red blood cell units within 4 hours from admission	
**Risk factor**	INR increase, Platelet count reduction	
		**Outcome**
		30-day mortality: 99 (29.9%)
**Centres**	1	**Variable: OR (95%-CI)**
**N° patients/centre/year**	90	Platelet count: 0.99 (0.99-0.99)
**Study duration (days)**	1338	INR: 1.43 (1.02-2.01)
**Total (included in the model)**	331	
	**GRADE CRITERIA**	
	**Statistical reporting**	Partial
	**Statistical quality**	Low
**Downgrading**	Appropriate eligibility criteria	Yes
	Measurement of exposure	Yes
	Measurement of outcome	Yes
	Adequate control for confounding	No
	Bias	very serious
**Up-grading**	Size of effect	Very large
	Residual confounding	Does not indicate upgrading
	Dose /response	Yes
	**DETAILS**	
**Downgrading**	Adequate control for confounding: Only 3 variables remained in the model an insufficient number for explanatory purposes. Statistical reporting: Logistic regression. Checking for conformity with linear gradient for continuos variables not reported. Test for interaction not reported. Goodness-of-fit assessment not reported. Collinearity assessment not reported. Statistical tests for models not reported. Variable selection method: Stepwise backward elimination. Reporting of variable coding method not indicated. Statistical quality: Possibly underfitted of the model.	
**Up-grading**	Size of effect: The 43% mortality risk with each unitary INR increase was generated by a potentially biased model. Upgrading not indicated.	
**External validity**	Single centre study	
**Conclusive evaluation**	GRADE rating up/down	Downgraded study
	GRADE rating	Very low evidence
	Statistical reporting	Partial
	Statistical quality	Low
	External validity issues	Yes
	Final grading	Downgraded study
	Final level of evidence	Very low evidence

Table S1 (continued from the previous page)

Observational study	4	Very low evidence - Downgraded study
**Year**	2003	
**Journal**	JT	
**First Author**	MacLeod	
**Statistical method**	Logistic regression	
**Inclusion criteria**	All patients with trauma	
**Risk factor**	PT and APTT increase	
		**Outcome**
		Hospital mortality: NA
**Centres**	1	**Variable: OR (95%-CI)**
**N° patients/centre/year**	1272	PT: 1.35 (1.11-1.68)
**Study duration (days)**	2191	APTT: 4.26 (3.23-5.62)
**Total (included in the model)**	7638	
	**GRADE CRITERIA**	
	**Statistical reporting**	Partial
	**Statistical quality**	Low
**Downgrading**	GRADE overall	Yes
	Indirectness	Yes
	Imprecision	Yes
	Other	No
	Publication bias	very serious
**Up-grading**	Size of effect	Large
	Residual confounding	Does not indicate upgrading
	Dose /response	Yes
	**DETAILS**	
**Downgrading**	Adequate control for confounding: Insufficient number of variables for an explanatory model, variables were arbitrarily dichotomized. Statistical reporting: Logistic regression. No Statistical support reported. Checking for conformity with linear gradient for continuos variables not reported. Dichotomization of continuos variables. Test for interaction not reported. Goodness-of-fit assessment not reported. Collinearity assessment not reported. Statistical tests for models not reported. Variable selection method: Not Reported. Reporting of variable coding method not performed. Comments: Crude mortality rate of patients included in logistic regression not available. There were 1276 deaths out of 14397 patients in the overall cohort (8.9%). Statistical quality: Dichotomization of almost all continuos variables included in the model was not justified and thus was probably based on arbitrary cut-offs. The model did not include important predictors.	
**Up-grading**	Size of effect: Large effect for PTT, generated however by a potentially biased model. Dose /response: FFP:PRBC ratio significant as a continuous variable generated, however, by a probably biased model. Upgrading not indicated.	
**External validity**	Single centre study	
**Conclusive evaluation**	GRADE rating up/down	Downgraded study
	GRADE rating	Very low evidence
	Statistical reporting	Partial
	Statistical quality	Low
	External validity issues	No
	Final grading	Downgraded study
	Final level of evidence	Very low evidence

Table S1 (continued from the previous page)

Observational study	5	Very low evidence - Downgraded study
**Year**	2011	
**Journal**	JT	
**First Author**	Sambasivan	
**Statistical method**	Logistic regression	
**Inclusion criteria**	Patients receiving at least one but less than 10 PRBC units within 24 hours from admission	
**Risk factor**	APTT increase	
		**Outcome**<br>Hospital mortality: 173 (14.6%)
**Centres**	23	**Variable: OR (95%-CI)**
**N° patients/centre/year**	22	APTT: 1.015 (1.010-1.019)
**Study duration (days)**	851	
**Total (included in the model)**	1181	
	**GRADE CRITERIA**	
	**Statistical reporting**	Partial
	**Statistical quality**	Low
**Downgrading**	GRADE overall	Yes
	Indirectness	Yes
	Imprecision	Yes
	Other	No
	Publication bias	very serious
**Up-grading**	Size of effect	Very large
	Residual confounding	Does not indicate upgrading
	Dose /response	Yes
	**DETAILS**	
**Downgrading**	Adequate control for confounding: Only 4 variables remained in the model an insufficient number for explanatory purposes. Statistical reporting: Proportional hazards including propensity score. Checking for conformity with linear gradient for continuos variables not reported. Test for interaction not reported. Goodness-of-fit assessment not reported. Collinearity assessment not reported. Statistical tests for models not reported. Variable selection method: Stepwise forward selection. Reporting of variable coding method not indicated. Statistical quality: Hazard proporionals assumption not checked. Possible underfitting of the model.	
**Up-grading**	Size of effect: Very large protective effect generated, however, by a potentially biased model. No upgrading indicated. Dose /response: FFP:PRBC ratio significant as a continuous variable generated, however, by a probably biased model. Upgrading not indicated.	
**External validity**	Multicentre study, with sufficient number of patients per centre	
**Conclusive evaluation**	GRADE rating up/down	Downgraded study
	GRADE rating	Very low evidence
	Statistical reporting	Partial
	Statistical quality	Low
	External validity issues	Yes
	Final grading	Downgraded study
	Final level of evidence	Very low evidence
