# Supplementary material for: Blood Component Therapy and Coagulopathy in Trauma: A Systematic Review of the Literature from the Trauma Update Group
Source: PLoS One. 2016 Oct 3;11(10):e0164090. doi: 10.1371/journal.pone.0164090 (PMC5047588; doi:10.1371/journal.pone.0164090)

S2-file: MEDLINE database search, flow diagram illustrating the literature selection process, and evidence assessment for the first query and results from the PROPPR trial.

Query # 2: *"Does a fixed blood-plasma transfusion ratio reduce mortality in trauma?"*

PubMed search details: search date December 14 2014

(((("blood transfusion"[MeSH Terms] OR ("blood"[All Fields] AND "transfusion"[All Fields]) OR "blood transfusion"[All Fields] OR "transfusion"[All Fields]) AND ("policy"[MeSH Terms] OR "policy"[All Fields])) OR (("blood transfusion"[MeSH Terms] OR ("blood"[All Fields] AND "transfusion"[All Fields]) OR "blood transfusion"[All Fields] OR "transfusion"[All Fields]) AND strategy[All Fields]) OR (("blood transfusion"[MeSH Terms] OR ("blood"[All Fields] AND "transfusion"[All Fields]) OR "blood transfusion"[All Fields] OR "transfusion"[All Fields]) AND ("Ratio (Oxf)"[Journal] OR "ratio"[All Fields])) OR (massive[All Fields] AND ("blood transfusion"[MeSH Terms] OR ("blood"[All Fields] AND "transfusion"[All Fields]) OR "blood transfusion"[All Fields] OR "transfusion"[All Fields]))) AND (("injuries"[Subheading] OR "injuries"[All Fields] OR "trauma"[All Fields] OR "wounds and injuries"[MeSH Terms] OR ("wounds"[All Fields] AND "injuries"[All Fields]) OR "wounds and injuries"[All Fields]) OR (traumatic[All Fields] AND ("haemorrhage"[All Fields] OR "hemorrhage"[MeSH Terms] OR "hemorrhage"[All Fields])) OR ("shock, traumatic"[MeSH Terms] OR ("shock"[All Fields] AND "traumatic"[All Fields]) OR "traumatic shock"[All Fields] OR ("traumatic"[All Fields] AND "shock"[All Fields])))) AND ("2000"[PDAT] : "3000"[PDAT])

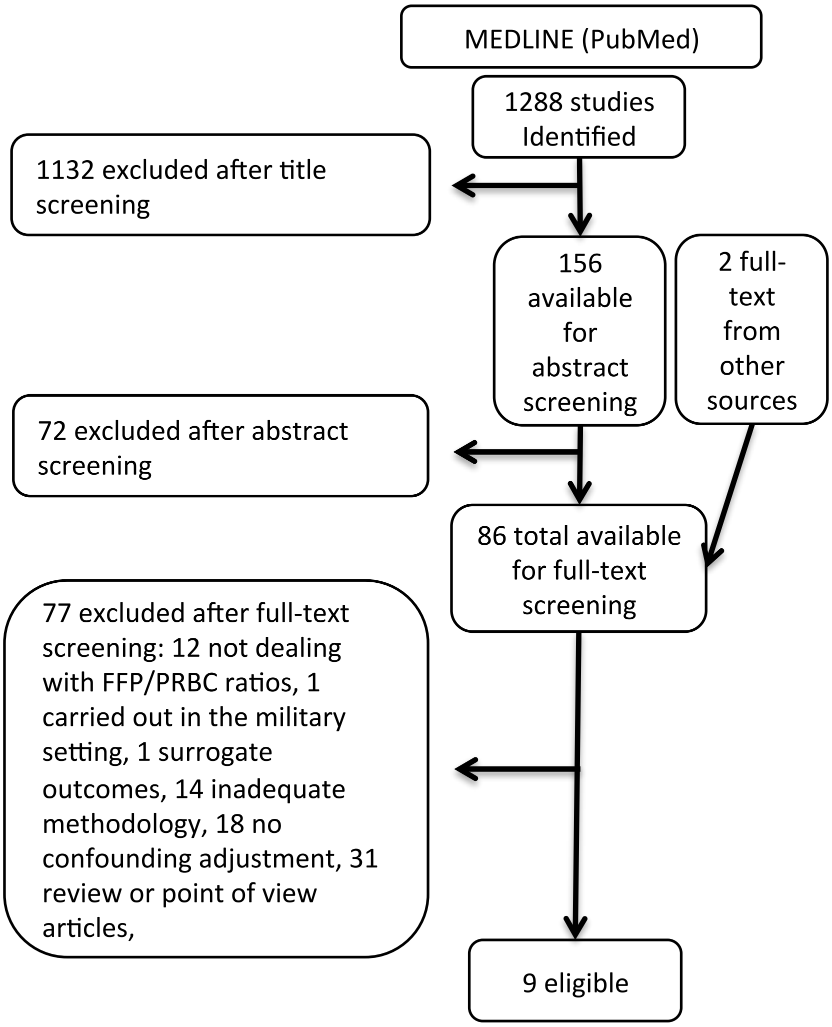
MEDLINE (PubMed)
1288 studies Identified
1132 excluded after title screening
156 available for abstract screening
2 full-text from other sources
72 excluded after abstract screening
86 total available for full-text screening
77 excluded after full-text screening: 12 not dealing with FFP/PRBC ratios, 1 carried out in the military setting, 1 surrogate outcomes, 14 inadequate methodology, 18 no confounding adjustment, 31 review or point of view articles,
9 eligible

Table S2

Observational study	1	
**Year**	2008	
**Journal**	AS	
**First Author**	Scalea	
**Statistical method**	Logistic regression	
**Inclusion criteria**	Patients admitted to the ICU for trauma occured within 24 hours	
**Treatment**	PRBC:FFP ratio as a continuous variable or 1:1	
		**Outcome**
		NA
**Centres**	1	**Variable: OR (95%-CI)**
**N° patients/centre/year**	NA	PRBC:FFP ratio 1:1: 0.57 (0.19-1.66)
**Study duration (days)**	882	PRBC:FFP ratio (continuous variable): 1.23 (0.81-1.87)
**Total (included in the model)**	NA	
	**GRADE CRITERIA**	
	**Statistical reporting**	Partial
	**Statistical quality**	Low
**Downgrading**	Appropriate eligibility criteria	Yes
	Measurement of exposure	Yes
	Measurement of outcome	Yes
	Adequate control for confounding	No
	Bias	very serious
	**GRADE overall**	
**Up-grading**	Size of effect	Not relevant
	Residual confounding	Does not indicate upgrading
	Dose /response	Yes
	**DETAILS**	
**Downgrading**	Adequate control for confounding: Important predictors were not included in the mortality model. Statistical reporting : Logistic regression. No Statistical support reported. Checking for conformity with linear gradient for continuos variables not reported. Test for interaction not reported. Goodness-of-fit assessment not reported. Collinearity assessment not reported. Statistical tests for models not reported. Reporting of variable coding method not indicated. Comments: 365 patients received PRBC, and of these 250 also FFP (thus, entering the logistic regression models). Statistical quality: Bivariate analysis for variable selection is an inappropriate method, especially when dealing with small samples. Automatic procedures such as the stepwise procedure used in the study are also not the best choice.The model was overfitted since it included 8 variables (not more than 51 deaths occured). The ICU length of stay was included in the model introducing a bias. The study did not account for survival bias. No propensity score was developed.	
**Up-grading**		
**External validity**	Single center study. Number of patients used for logistic regression not clearly reported	
**Conclusive evaluation**	GRADE rating up/down	Downgraded study
	GRADE rating	Very low evidence
	Statistical reporting	Partial
	Statistical quality	Low
	External validity issues	Yes
	Final grading	Downgraded study
	Final level of evidence	Very low evidence

Table S2 (continued from the previous page)

Observational study	2	
**Year**	2010	
**Journal**	JACS	
**First Author**	Inaba	
**Statistical method**	Propensity score matching	
**Inclusion criteria**	Trauma admitted to surgical ICU receiving < 10 PRBC units within 12 hours from admssion (excluding deaths occured within 24 hours)	
**Treatment**	Receiving FFP	
		**Outcome**
		Hospital mortality: 89 (15.7%)
**Centres**	1	**Variable: OR (95%-CI)**
**N° patients/centre/year**	95	FFP: 1.27 (0.81-2.0)
**Study duration (days)**	2191	
**Total (included in the model)**	568	
**Hospital mortality**	89 (15.7%)	
	**GRADE CRITERIA**	
	**Statistical reporting**	Partial
	**Statistical quality**	Low
**Downgrading**	Appropriate eligibility criteria	Yes
	Measurement of exposure	Yes
	Measurement of outcome	Yes
	Adequate control for confounding	No
	Bias	very serious
	GRADE overall	
**Up-grading**	Size of effect	Not relevant
	Residual confounding	Does not indicate upgrading
	Dose /response	Not applicable
	**DETAILS**	
**Downgrading**	Adequate control for confounding: Important predictors were not included in the propensity score. Cut-offs for variable dichotomization were arbitrarily defined. Statistical reporting: The propensity score development process was not reported and was not available for quality assessment. Statistical quality: Insufficient statistical reporting. All continuous variables were dichotomized according to arbitrary cut-offs. Although the study was well designed from a statistical point of view, the lack of reporting of the propensity score limits its evaluability. The study did not account for survival bias.	
**Up-grading**		
**External validity**	Single center study	
**Conclusive evaluation**	GRADE rating up/down	Downgraded study
	GRADE rating	Very low evidence
	Statistical reporting	Partial
	Statistical quality	Low
	External validity issues	Yes
	Final grading	Downgraded study
	Final level of evidence	Very low evidence

Table S2 (continued from the previous page)

Observational	3		
**Year**	2011		
**Journal**	JT		
**First Author**	Wafaisade		
**Statistical method**	Logistic regression		
**Inclusion criteria**	Patients receiving more than 3 and less than 10 PRBC units from arrival to the ER and admission to the ICU (excluding those dying within one hour from hospital admission)		
**Treatment**	FFP:PRBC ratio > 1:1		
		**Outcome**<br>Hospital mortality: 321 (23.6%)	
**Centres**	116	**Variable: OR (95%-CI)**	
**N° patients/centre/year**	3	FFP:PRBC ratio <1:1: Ref = 1	
**Study duration (days)**	1460	FFP:PRBC ratio =1:1: 0.8 (0.54-1.18)	
**Total (included in the model)**	1362	FFP:PRBC ratio >1:1: 0.52 (0.31-0.87)	
	**GRADE CRITERIA**		
	**Statistical reporting**	Partial	
	**Statistical quality**	Low	
**Downgrading**	Appropriate eligibility criteria	0	
	Measurement of exposure	No	
	Measurement of outcome	No	
	Adequate control for confounding	No	
	Bias	Not assessable	
	GRADE overall	Not assessable	
**Up-grading**	Size of effect	Not relevant	
	Residual confounding	Does not indicate upgrading	
	Dose /response	Yes	
	**DETAILS**		
**Downgrading**	Adequate control for confounding: Only 3 variables remained in the model an insufficient number for explanatory purposes. Statistical reporting: Logistic regression. No Statistical support reported. Checking for conformity with linear gradient for continuos variables not reported. Test for interaction not reported. Goodness-of-fit assessment not reported. Collinearity assessment not reported. Statistical tests for models not reported. Variable selection method: Stepwise forward selection. Reporting of variable coding method not performed.. Statistical quality: Insufficient statistical reporting. Bivariate analysis was performed to select variables to enter the stepwise forward selection process, using a low p value cut-off (0.05). Total-body CT scan surprisingly turned out to be protective, the study did not account for survival bias. No propensity score was developed though a treatment was investigated.		
**Up-grading**	Dose /response: Progressive Odds Ratios reduction with increasing PRBC:FFP ratios generated , however, by a potentially biased model. Upgrading not indicated.		
**External validity**	Only 3 patients/centre/year were recruited on average questioning the representativeness of the sample.		
**Conclusive evaluation**	GRADE rating up/down	Downgraded study	
	GRADE rating	Very low evidence	
	Statistical reporting	Partial	
	Statistical quality	Low	
	External validity issues	Yes	
	Final grading	Downgraded study	
	Final level of evidence	Very low evidence	

Table S2 (continued from the previous page)

Observational study	4	
**Year**	2013	
**Journal**	JAMA surg	
**First Author**	Holcomb	
**Statistical method**	Cox proportional hazards	
**Inclusion criteria**	Patients requiring the highest level of trauma activation receiving and at least one PRBC unit within 6 hours from admission	
**Treatment**	FFP:PRBC ratio >= 1:1 received between 30 minutes and 6 hours from admission	
		**Outcome**
		#VALUE!
**Centres**	10	**Variable: OR (95%-CI)**
**N° patients/centre/year**	79	FFP:PRBC ratio ≥ 1:1: HR 0.23 (95%-CI NA)
**Study duration (days)**	406	FFP:PRBC ratio: ≥ 1:2-<1:1: HR 0.42 (95%-CI NA)
**Total (included in the model)**	876	FFP:PRBC ratio < 1:2: HR ref=1 (95%-CI NA)
		FFP:PRBC (continuous): HR 0.31 (0.16-0.58)
	**GRADE CRITERIA**	
	**Statistical reporting**	Sufficient for quality assessment
	**Statistical quality**	High
**Downgrading**	GRADE overall	0
	Indirectness	No
	Imprecision	No
	Other	No
	Publication bias	Not assessable
	Inconsistency with other studies	Not assessable
**Up-grading**	Size of effect	Large
	Residual confounding	Does not indicate upgrading
	Dose /response	Yes
	**DETAILS**	
**Downgrading**	Adequate control for confounding: Insufficient number of variables for an explanatory model. Statistical reporting: Multi-level time-dependent Cox proportional hazards regression. Dichotomization of continuos variables. Goodness-of-fit assessment not reported. Collinearity assessment not reported. Statistical tests for models not reported. Variable selection method: Purposeful variables selection strategy. Reporting of variable coding method not performed. Statistical quality: Sophisticated analysis accounting for survival bias. However, propensity score for different FFP-PRBC ratio approaches would have been indicated but was not performed, potentially generating a selection bias.	
**Up-grading**	Size of effect: Large protective effect generated, however, by a potentially biased model. No upgrading indicated. Dose /response: FFP:PRBC ratio significant as a continuous variable generated, however, by a probably biased model. Upgrading not indicated.	
**External validity**	Multicenter study, with an adequate number of patients per center	
**Conclusive evaluation**	GRADE rating up/down	No grading modification
	GRADE rating	Low evidence
	Statistical reporting	Sufficient for quality assessment
	Statistical quality	High
	External validity issues	No
	Final grading	No grading modification
	Final level of evidence	Low evidence

Table S2 (continued from the previous page)

Observational study	5	
**Year**	2009	
**Journal**	JT	
**First Author**	Teixeira	
**Statistical method**	Logistic regression	
**Inclusion criteria**	Trauma patients receiving 10 or more PRBC units within the first 24 hours	
**Treatment**	FFP:PRBC ratio (continuous variable)	
		**Outcome**<br>Hospital mortality: 161 (42%)
**Centres**	1	**Variable: OR (95%-CI)**
**N° patients/centre/year**	64	FFP:PRBC ratio: 0.02 (0.01-0.07)
**Study duration (days)**	2191	
**Total (included in the model)**	383	
	**GRADE CRITERIA**	
	**Statistical reporting**	Partial
	**Statistical quality**	Low
**Downgrading**	GRADE overall	0
	Indirectness	No
	Imprecision	No
	Other	No
	Publication bias	Not assessable
	Inconsistency with other studies	Not assessable
**Up-grading**	Size of effect	Very large
	Residual confounding	Does not indicate upgrading
	Dose /response	Yes
	**DETAILS**	
**Downgrading**	Adequate control for confounding: Important predictors were not included in the propensity score. Cut-offs for variable dichotomization were arbitrarily defined. Statistical reporting: Logistic regression. No Statistical support reported. Checking for conformity with linear gradient for continuos variables not reported. Test for interaction not reported. Goodness-of-fit assessment not reported. Collinearity assessment not reported. Variable selection method: Stepwise bidirectional elimination after bivariate selection. Reporting of variable coding method not performed. Statistical quality: Insufficient statistical reporting. All continuous variables were arbitrarily dichotomized with the exception of the FFP:PRBC Bivariate variables selection before automatic procedure (stepwise bidirectional elimination). The study did not account for survival bias. No propensity score was developed.	
**Up-grading**	Size of effect: Very large protective effect generated, however, by a potentially biased model. No upgrading indicated. Dose /response: FFP:PRBC ratio significant as a continuous variable generated, however, by a probably biased model. Upgrading not indicated.	
**External validity**	Single center study.	
**Conclusive evaluation**	GRADE rating up/down	Downgraded study
	GRADE rating	Very low evidence
	Statistical reporting	Partial
	Statistical quality	Low
	External validity issues	Yes
	Final grading	Downgraded study
	Final level of evidence	Very low evidence

Table S2 (continued from the previous page)

Observational	6	
**Year**	2011	
**Journal**	JT	
**First Author**	Sambavisan	
**Statistical method**	Cox proportional hazards including propensity score	
**Inclusion criteria**	Patients receiving at least one but less than 10 PRBC units within 24 hours from admission (excluding deaths occured within 2 hours)	
**Treatment**	FFP:PRBC ratio ≥1	
		**Outcome**<br>Hospital mortality: 173 (14.6%)
**Centres**	23	**Variable: OR (95%-CI)**
**N° patients/centre/year**	22	FFP:PRBC ratio ≥1: HR 0.87 (0.55-1.38)
**Study duration (days)**	851	
**Total (included in the model)**	1181	
	**GRADE CRITERIA**	
	**Statistical reporting**	Partial
	**Statistical quality**	Low
**Downgrading**	GRADE overall	0
	Indirectness	No
	Imprecision	No
	Other	No
	Publication bias	Not assessable
	Inconsistency with other studies	Not assessable
**Up-grading**	Size of effect	Not relevant
	Residual confounding	Does not indicate upgrading
	Dose /response	Not applicable
	**DETAILS**	
**Downgrading**	Adequate control for confounding: Only five variables remained in the final model, insufficient number for explanatory purposes. Few variables were also included in the propensity score. Statistical reporting: Proportional hazards including propensity score. Checking for conformity with linear gradient for continuos variables not reported. Test for interaction not reported. Goodness-of-fit assessment not reported. Collinearity assessment not reported. Statistical tests for models not reported. Variable selection method: Stepwise forward selection. Reporting of variable coding method not indicated. Statistical quality: Insufficient statistical reporting. Hazard proportional assumption not checked. Few variables entered the mortality model and important covariates were not considered. Low statistical quality. The study did not account for survival bias.	
**Up-grading**		
**External validity**	Multicenter study, with an acceptable number of patients treated per center.	
**Conclusive evaluation**	GRADE rating up/down	Downgraded study
	GRADE rating	Very low evidence
	Statistical reporting	Partial
	Statistical quality	Low
	External validity issues	Yes
	Final grading	Downgraded study
	Final level of evidence	Very low evidence

Table S2 (continued from the previous page)

Observational	7		
**Year**	2011		
**Journal**	JT		
**First Author**	Holcomb		
**Statistical method**	Cox proportional hazards including propensity score		
**Inclusion criteria**	Trauma patients receiving 10 or more PRBC units within 24 hours from admission		
**Treatment**	FFP:PRBC ratio (continuous variable)		
		**Outcome**	
		30-day mortality: 181 (28.1%)	
**Centres**	22	**Variable: OR (95%-CI)**	
**N° patients/centre/year**	29	FFP:PRBC ratio: HR 0.49 (0.28-0.86)	
**Study duration (days)**	364		
**Total (included in the model)**	643		
	**GRADE CRITERIA**		
	**Statistical reporting**	Partial	
	**Statistical quality**	Low	
**Downgrading**	GRADE overall	0	
	Indirectness	No	
	Imprecision	No	
	Other	No	
	Publication bias	Not assessable	
	Inconsistency with other studies	Not assessable	
**Up-grading**	Size of effect	Large	
	Residual confounding	Does not indicate upgrading	
	Dose /response	Yes	
	**DETAILS**		
**Downgrading**	Adequate control for confounding: Few variables included in the final model, insufficient for explanatory purposes. Statistical reporting: Statistical model: Cox proportional hazards. No Statistical support reported. Checking for conformity with linear gradient for continuos variables not reported. Test for interaction not reported. Goodness-of-fit assessment not reported. Collinearity assessment not reported. Statistical tests for models not reported. Variable selection method: Not Reported. Reporting of variable coding method not performed. Statistical quality: Insufficient statistical reporting. Important covariates (e.g. age) were not included in the mortality model (underfitting). No propensity score for FFP:PRBC ratio was developed. The study did not account for survival bias.		
**Up-grading**	Size of effect: Large protective effect generated, however, by a potentially biased model. No upgrading indicated. Dose /response: FFP:PRBC ratio significant as a continuous variable generated, however, by a probably biased model. Upgrading not indicated.		
**External validity**	Multicenter study, with an acceptable number of patients treated per center.		
**Conclusive evaluation**	GRADE rating up/down	Downgraded study	
	GRADE rating	Very low evidence	
	Statistical reporting	Partial	
	Statistical quality	Low	
	External validity issues	No	
	Final grading	Downgraded study	
	Final level of evidence	Very low evidence	

Table S2 (continued from the previous page)

Observational	8	
**Year**	2011	
**Journal**	VS	
**First Author**	Borgman	
**Statistical method**	Logistic regression	
**Inclusion criteria**	TASH score ≥ 15 excluding patients died within 1 hour from admission	
**Treatment**	FFP:PRBC ratio (continuous variable)	
		**Outcome**<br>Hospital mortality: NA (NA%)
**Centres**	100	**Variable: OR (95%-CI)**
**N° patients/centre/year**	1	FFP:PRBC ratio: Survival OR 2.5 (1.56-4.00)
**Study duration (days)**	2190	
**Total (included in the model)**	557	
	**GRADE CRITERIA**	
	**Statistical reporting**	Partial
	**Statistical quality**	Low
**Downgrading**	GRADE overall	0
	Indirectness	No
	Imprecision	No
	Other	No
	Publication bias	Not assessable
	Inconsistency with other studies	Not assessable
**Up-grading**	Size of effect	Large
	Residual confounding	Does not indicate upgrading
	Dose /response	Yes
	**DETAILS**	
**Downgrading**	Adequate control for confounding: Only three variables remained in the final model, insufficient number for explanatory purposes. Few variables were also included in the propensity score. Statistical reporting: Statistical model: Logistic regression. No Statistical support reported. Checking for conformity with linear gradient for continuos variables not reported. Test for interaction not reported. Internal validity assessment not reported. Goodness-of-fit assessment not reported. Collinearity assessment not reported. Statistical tests for models not reported. Reporting of variable coding method not indicated. Statistical quality: Insufficient statistical reporting. No propensity score for treatment performed. Variable selection method: Bivariate analysis. Only three variables entered the model that was clearly underfitted. no propensity score for FFP:PRBC ratio was developed.	
**Up-grading**	Size of effect: Large protective effect generated, however, by a potentially biased model. No upgrading indicated. Dose /response: FFP:PRBC ratio significant as a continuous variable generated, however, by a probably biased model. Upgrading not indicated.	
**External validity**	Multicenter study, with 1 patients admitted per center on average. Probably several centers did not enroll patients. Inadequate reporting.	
**Conclusive evaluation**	GRADE rating up/down	Downgraded study
	GRADE rating	Very low evidence
	Statistical reporting	Partial
	Statistical quality	Low
	External validity issues	Yes
	Final grading	Downgraded study
	Final level of evidence	Very low evidence

Table S2 (continued from the previous page)

Observational	9	
**Year**	2010	
**Journal**	Injury	
**First Author**	Mitra	
**Statistical method**	Logistic regression	
**Inclusion criteria**	Patients receiving more than 4 packed red blood cell units within 4 hours from admission	
**Treatment**	FFP:PRBC ratio measured at 4 hours from admission (continuous variable)	
		**Outcome**<br>30-day mortality: 99 (29.9%)
**Centres**	1	**Variable: OR (95%-CI)**
**N° patients/centre/year**	90	FFP:PRBC ratio: 0.15 (0.05-0.48)
**Study duration (days)**	1338	
**Total (included in the model)**	331	
	**GRADE CRITERIA**	
	**Statistical reporting**	Partial
	**Statistical quality**	Low
**Downgrading**	GRADE overall	0
	Indirectness	No
	Imprecision	No
	Other	No
	Publication bias	Not assessable
	Inconsistency with other studies	Not assessable
**Up-grading**	Size of effect	Very large
	Residual confounding	Does not indicate upgrading
	Dose /response	Yes
	**DETAILS**	
**Downgrading**	Adequate control for confounding: Only five variables remained in the final model, insufficient number for explanatory purposes. Few variables were also included in the propensity score. Statistical reporting: Logistic regression. Checking for conformity with linear gradient for continuos variables not reported. Test for interaction not reported. Internal validity assessment not reported. Goodness-of-fit assessment not reported. Collinearity assessment not reported. Statistical tests for models not reported. Variable selection method: Stepwise backward elimination. Reporting of variable coding method not indicated. Statistical quality: Insufficient statistical reporting. Possible underfitting of the model, no propensity score for FFP administraton was developed. The study did not account for survival bias.	
**Up-grading**	Size of effect: Large protective effect generated, however, by a potentially biased model. No upgrading indicated. Dose /response: FFP:PRBC ratio significant as a continuous variable generated, however, by a probably biased model. Upgrading not indicated.	
**External validity**	Single center study	
**Conclusive evaluation**	GRADE rating up/down	Downgraded study
	GRADE rating	Very low evidence
	Statistical reporting	Partial
	Statistical quality	Low
	External validity issues	Yes
	Final grading	Downgraded study
	Final level of evidence	Very low evidence

Table S2 (continued from the previous page)

RCT 1				
Year	2015	First Author	Holcomb	
Journal	JAMA			
Sample	trauma patients for whom the highest level of activation was required			
Treatment	FFP/Platelets/PRBC ratio 1:1:1			
Control	FFP/Platelets/PRBC ratio 1:1:2			
Outcome	24-hour mortality			
			Outcome	
	n° pz	n		%
Treatment	335	43		12.8
Control	341	58		17.0
Total	676	101		14.9
Centres	12 Centres			
Power	0.332	TB 24 (95%-CI NNTB 10 to ∞ to NNTH 82)		
		GRADE CRITERIA		
Downgrading		Allocation concealment		Yes
		Intention to treat principle observed		Yes
		Blinding		No
		Completement of follow-up		Yes
		Early stopping		Yes
		Bias		No
		Indirectness		No
		Imprecision		No
		Publication bias		No
		Inconsistency with other trials		Not assessable
Up-grading		Size of effect		Not relevant
		Residual confounding		Not assessable
		Dose /response		Not relevant
		DETAILS		
Downgrading				
Up-grading				
**Conclusive evaluation**		GRADE rating up/down	No grading modification	
		GRADE rating	High evidence	
		Statistical reporting	Sufficient for quality assessment	
		Methodological and statistical quality	High	
		External validity issues	Yes	
		Final grading	No grading modification	
		Final level of evidence	High evidence	

Table S2 (continued from the previous page)

RCT 2			
Year	2015	First Author	Holcomb
Journal	JAMA		
Sample	trauma patients for whom the highest level of activation was required		
Treatment	FFP/Platelets/PRBC ratio 1:1:1		
Control	FFP/Platelets/PRBC ratio 1:1:2		
Outcome	30-day mortality		
		Outcome	
	n° pz	n	%
Treatment	335	75	22.4
Control	341	89	26.1
Total	676	164	24.3
Centres	Single Center		
Power	0.202	TB 27 (95%-CI NNTB 10 to ∞ to NNTH 36)	
		GRADE CRITERIA	
Downgrading		Allocation concealment	Yes
		Intention to treat principle observed	Yes
		Blinding	Yes
		Completement of follow-up	Yes
		Early stopping	No
		Statistical reporting	Sufficient for quality assessment
		Methodological and statistical quality	High
		Indirectness	No
		Publication bias	No
		Inconsistency with other trials	Not assessable
Up-grading		Size of effect	Not relevant
		Residual confounding	Not assessable
		Dose /response	Not relevant
		DETAILS	
Downgrading			
Up-grading			
**Conclusive evaluation**		GRADE rating up/down	No grading modification
		GRADE rating	High evidence
		Statistical reporting	Sufficient for quality assessment
		Methodological and statistical quality	High
		External validity issues	No
		Final grading	No grading modification
		Final level of evidence	High evidence

Figure S2 : Forest plots illustrating absolute and relative risks for the PROPPR trial. The 24-hour and 30-day mortality outcomes are reported

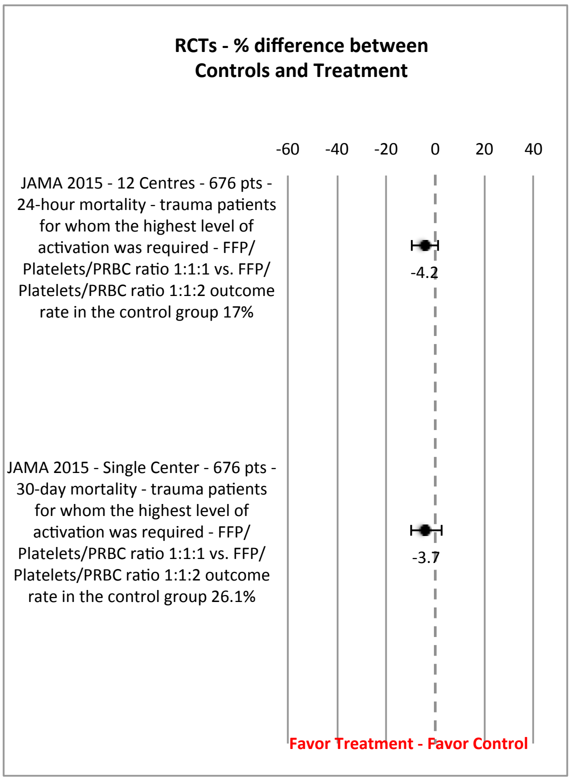

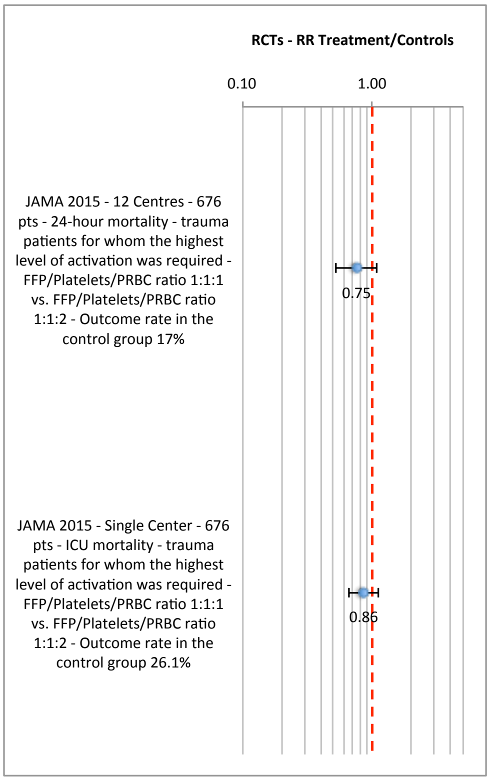

Supplement: S2 File — (PDF) [file pone.0164090.s002.pdf]
