# Supplementary material for: Blood Component Therapy and Coagulopathy in Trauma: A Systematic Review of the Literature from the Trauma Update Group
Source: PLoS One. 2016 Oct 3;11(10):e0164090. doi: 10.1371/journal.pone.0164090 (PMC5047588; doi:10.1371/journal.pone.0164090)
Supplement: S3 File — (PDF) [file pone.0164090.s003.pdf]

S3-file: MEDLINE database search, flow diagram illustrating the literature selection process, and evidence assessment for the first query.

Query # 3: *"Does hypofibrinogenemia treatment reduce mortality in trauma?"*

PubMed search details: search date December 13 2014

(((("fibrinogen"[MeSH Terms] OR "fibrinogen"[All Fields]) AND ("organization and administration"[MeSH Terms] OR ("organization"[All Fields] AND "administration"[All Fields]) OR "organization and administration"[All Fields] OR "administration"[All Fields])) OR (("fibrinogen"[MeSH Terms] OR "fibrinogen"[All Fields]) AND ("therapy"[Subheading] OR "therapy"[All Fields] OR "treatment"[All Fields] OR "therapeutics"[MeSH Terms] OR "therapeutics"[All Fields])) OR ("fibrinogen"[MeSH Terms] OR "fibrinogen"[All Fields])) AND (("injuries"[Subheading] OR "injuries"[All Fields] OR "trauma"[All Fields] OR "wounds and injuries"[MeSH Terms] OR ("wounds"[All Fields] AND "injuries"[All Fields]) OR "wounds and injuries"[All Fields]) OR (traumatic[All Fields] AND ("haemorrhage"[All Fields] OR "hemorrhage"[MeSH Terms] OR "hemorrhage"[All Fields])) OR ("shock, traumatic"[MeSH Terms] OR ("shock"[All Fields] AND "traumatic"[All Fields]) OR "traumatic shock"[All Fields] OR ("traumatic"[All Fields] AND "shock"[All Fields])))) AND ("2000/"[PDAT] : "3000"[PDAT])

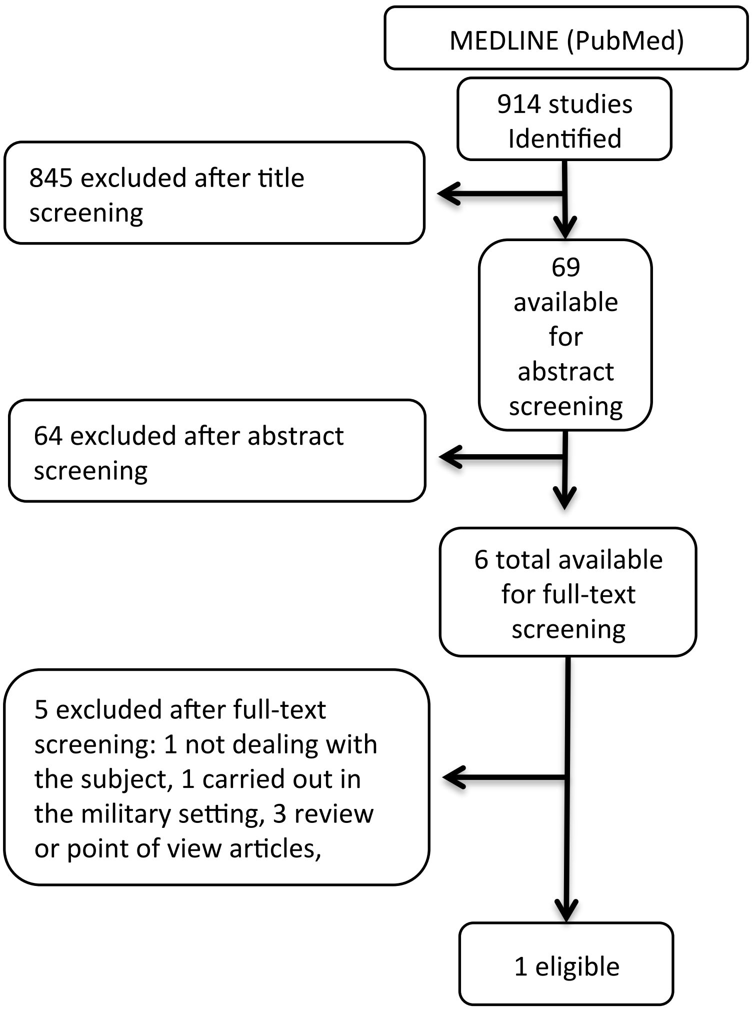
MEDLINE (PubMed)
914 studies Identified
845 excluded after title screening
69 available for abstract screening
64 excluded after abstract screening
6 total available for full-text screening
5 excluded after full-text screening: 1 not dealing with the subject, 1 carried out in the military setting, 3 review or point of view articles,
1 eligible

Table S3

Observational study	1	
**Year**	2012	
**Journal**	JTH	
**First Author**	Rourke	
**Statistical method**	Logistic regression	
**Inclusion criteria**	Time fron injury to arrival within 120 minutes, SBP < 90 at admission, poor responsiveness to initial fluid administration	
**Treatment**	Fibrinogen administration within the first 12 hours	
		**Outcome**
		NA
**Centres**	2	**Variable: OR (95%-CI)**
**N° patients/centre/year**	NA	Fibrinogen: 0.91 (0.81-1.01)
**Study duration (days)**	1095	
**Total (included in the model)**	NR	
	**GRADE CRITERIA**	
	**Statistical reporting**	Partial
	**Statistical quality**	Low
**Downgrading**	Appropriate eligibility criteria	Yes
	Measurement of exposure	Yes
	Measurement of outcome	Yes
	Control for confounding	No
	Bias	very serious
	**GRADE overall**	
**Up-grading**	Size of effect	Very large
	Residual confounding	Does not indicate upgrading
	Dose /response	Yes
	**DETAILS**	
**Downgrading**	Control for confounding: Important predictors were not included in the mortality model. Statistical reporting : Subgroup of coagulopathic patients from a sample of 517 patients. Not reported the number of patients and of deaths. Logistic regression. No Statistical support reported. Checking for conformity with linear gradient for continuos variables not reported. Test for interaction not reported. Internal validity assessment not reported. Goodness-of-fit assessment not reported. Collinearity assessment not reported. Statistical tests for models not reported. Variable selection method: Stepwise forward selection. Reporting of variable coding method not performed. Statistical quality: Insufficient statistical reporting. Important covariates are missing in the model (possible underfitting). No propensity score was developed. The analysis does not account for survival bias.	
**Up-grading**		
**External validity**	Only two centres participating to the study	
**Conclusive evaluation**	GRADE rating up/down	Downgraded study
	GRADE rating	Very low evidence
	Statistical reporting	Partial
	Statistical quality	Low
	External validity issues	Yes
	Final grading	Downgraded study
