# Supplementary material for: Blood Component Therapy and Coagulopathy in Trauma: A Systematic Review of the Literature from the Trauma Update Group
Source: PLoS One. 2016 Oct 3;11(10):e0164090. doi: 10.1371/journal.pone.0164090 (PMC5047588; doi:10.1371/journal.pone.0164090)
Supplement: S5 File — (PDF) [file pone.0164090.s005.pdf]

Suggestions for future observational studies:

Definitions	1) Do not define bleeding severity according to the number of PBRC units transfused in a specific timeframe. Definition should be based on parameters collected on admission<br>2) Define coagulopathy
Case mix selection	1) Bleeding without coagulopathy<br>2) Bleeding with coagulopathy<br>3) Bleeding without coagulopathy and timely surgical/interventional haemostasis<br>4) Bleeding with coagulopathy and delayed surgical/interventional haemostasis<br>5) Terminal bleeding with consequent coagulopathy
Methodology and statistics	1) Dataset: high quality database collecting items predefined to address specific queries<br>2) Design: Availability of a control group (e.g. patients not receiving a specific treatment) for comparisons with the study group<br>3) Statistics: control for confounding using appropriate statistical models methods (e.g. logistic regression, propensity score matching)<br>4) Design and statistics: large cohorts should be analysed to avoid overfitting; at least 10 outcomes should be available for each variable included in the models (including the initial models before further variable selection)<br>5) Dataset and statistics: the number of variables should be sufficient to develop *explanatory* models (i.e. most known important predictors should be included), avoid underfitting<br>6) Statistics: avoid bivariate methods to select variables to be included in the model<br>7) Statistics: be cautious in the use of automatic selection procedures for covariate selection, particularly when the sample size is not large<br>8) Statistics: account for the immortal-time bias when dealing with time-dependent treatments<br>9) Statistics: account for treatment selection bias (balance the probability of receiving treatment between study and control group)
Treatment side effects	Collect data concerning potential side effects (e.g. ALI after transfusion, deep venous or thrombosis after fibrinogen administration, etc.)
Outcome	Besides short-term outcomes (such as 6-24 hours, 28-days) middle-long term outcomes should be preferred (at least hospital outcomes; 6-months outcomes could be indicated in specific conditions: elderly, head trauma)
External validation	Dataset: the number of patients and centres participating should be sufficiently large to allow the generalizability of the results
