# Supplementary material for: Blood Component Therapy and Coagulopathy in Trauma: A Systematic Review of the Literature from the Trauma Update Group
Source: PLoS One. 2016 Oct 3;11(10):e0164090. doi: 10.1371/journal.pone.0164090 (PMC5047588; doi:10.1371/journal.pone.0164090)
Supplement: S1 PRISMA Checklist — (PDF) [file pone.0164090.s006.pdf]

S5-file: In this file we report the Checklist of items included in our systematic review according to the Preferred Reporting Items for Systematic Reviews and Meta-Analyses (PRISMA) statement recommendations.

We considered the retrieved studies too heterogeneous particularly in terms of design to be combined in meta-analyses, which hence have not been performed. We also were unable to formally assess the publication risk of bias, because the number of study retrieved was insufficient to gain sufficient power for the test. We instead complied with all the other recommendations. The main limitation of the review was that we searched only the MEDLINE database using the free PubMed provider.

Table S5			
**Section/topic**	**#**	**Checklist item**	**Reported on page #**
**TITLE**			
Title	1	Identify the report as a systematic review, meta-analysis, or both.	Page 1
**ABSTRACT**			
Structured summary	2	Provide a structured summary including, as applicable: background; objectives; data sources; study eligibility criteria, participants, and interventions; study appraisal and synthesis methods; results; limitations; conclusions and implications of key findings; systematic review registration number.	Page 2
**INTRODUCTION**			
Rationale	3	Describe the rationale for the review in the context of what is already known.	First paragraph of the Introduction section
Objectives	4	Provide an explicit statement of questions being addressed with reference to participants, interventions, comparisons, outcomes, and study design (PICOS).	Second paragraph of the Introduction section
**METHODS**			
Protocol and registration	5	Indicate if a review protocol exists, if and where it can be accessed (e.g., Web address), and, if available, provide registration information including registration number.	Second paragraph of the Data Analysis subsection
Eligibility criteria	6	Specify study characteristics (e.g., PICOS, length of follow-up) and report characteristics (e.g., years considered, language, publication status) used as criteria for eligibility, giving	Study Selection subsection

Table S5			
**Section/topic**	**#**	**Checklist item**	**Reported on page #**
		rationale.	
Information sources	7	Describe all information sources (e.g., databases with dates of coverage, contact with study authors to identify additional studies) in the search and date last searched.	Literature Search Strategies subsection, S1-File, S2-file, S3-file, and S4-file
Search	8	Present full electronic search strategy for at least one database, including any limits used, such that it could be repeated.	S1-File, S2-file, S3-file, S4-file,
Study selection	9	State the process for selecting studies (i.e., screening, eligibility, included in systematic review, and, if applicable, included in the meta-analysis).	Study Selection subsection
Data collection process	10	Describe method of data extraction from reports (e.g., piloted forms, independently, in duplicate) and any processes for obtaining and confirming data from investigators.	Study Selection subsection, Data Analyses subsection
Data items	11	List and define all variables for which data were sought (e.g., PICOS, funding sources) and any assumptions and simplifications made.	S1-File, S2-file, S3-file, S4-file,
Risk of bias in individual studies	12	Describe methods used for assessing risk of bias of individual studies (including specification of whether this was done at the study or outcome level), and how this information is to be used in any data synthesis.	Evidence Grading subsection
Summary measures	13	State the principal summary measures (e.g., risk ratio, difference in means).	Data Analyses subsection
Synthesis of results	14	Describe the methods of handling data and combining results of studies, if done, including measures of consistency (e.g., I^2) for each meta-analysis.	Data Analyses subsection
Risk of bias across studies	15	Specify any assessment of risk of bias that may affect the cumulative evidence (e.g., publication bias, selective	Evidence Grading subsection

Table S5			
**Section/topic**	**#**	**Checklist item**	**Reported on page #**
		reporting within studies).	
Additional analyses	16	Describe methods of additional analyses (e.g., sensitivity or subgroup analyses, meta-regression), if done, indicating which were pre-specified.	No additional analyses were performed
**RESULTS**			
Study selection	17	Give numbers of studies screened, assessed for eligibility, and included in the review, with reasons for exclusions at each stage, ideally with a flow diagram.	Results section, S1-File, S2-file, S3-file, S4-file,
Study characteristics	18	For each study, present characteristics for which data were extracted (e.g., study size, PICOS, follow-up period) and provide the citations.	Results section, Tables 1, 2, 3, 4, 5. S1-File, S2-file, S3-file, S4-file,
Risk of bias within studies	19	Present data on risk of bias of each study and, if available, any outcome-level assessment (see Item 12).	Results section, S1-File, S2-file, S3-file, S4-file,
Results of individual studies	20	For all outcomes considered (benefits or harms), present, for each study: (a) simple summary data for each intervention group and (b) effect estimates and confidence intervals, ideally with a forest plot.	Results section, Tables 1, 2, 3, 4, 5. S1-File, S2-file, S3-file, S4-file,
Synthesis of results	21	Present results of each meta-analysis done, including confidence intervals and measures of consistency.	No meta-analysis was performed because studies were not sufficiently homogenous
Risk of bias across studies	22	Present results of any assessment of risk of bias across studies (see Item 15).	Formal assessment not applicable because of the insufficient number of studies available.
Additional analysis	23	Give results of additional analyses, if done (e.g., sensitivity or subgroup analyses, meta-regression [see Item 16]).	No additional analyses were performed.
**DISCUSSION**			

**Table S5**			
**Section/topic**	**#**	**Checklist item**	**Reported on page #**
Summary of evidence	24	Summarize the main findings including the strength of evidence for each main outcome; consider their relevance to key groups (e.g., health care providers, users, and policy makers).	Results section, Tables 1, 2, 3, 4, 5. S1-File, S2-file, S3-file, S4-file,
Limitations	25	Discuss limitations at study and outcome level (e.g., risk of bias), and at review level (e.g., incomplete retrieval of identified research, reporting bias).	Results section, S1-file, S4-file.<br>Study Limitations subsection
Conclusions	26	Provide a general interpretation of the results in the context of other evidence, and implications for future research.	Results section
**FUNDING**			
Funding	27	Describe sources of funding for the systematic review and other support (e.g., supply of data); role of funders for the systematic review.	Funding section at the end of the paper
